# Geographic range size and speciation in honeyeaters

**DOI:** 10.1186/s12862-022-02041-6

**Published:** 2022-06-29

**Authors:** Eleanor M. Hay, Matthew D. McGee, Steven L. Chown

**Affiliations:** School of Biological Sciences, Monash University, Melbourne, VIC 3800 Australia

**Keywords:** Allopatry, Islands, Macroecology, Meliphagidae, Speciation mode, Geographic range

## Abstract

**Background:**

Darwin and others proposed that a species’ geographic range size positively influences speciation likelihood, with the relationship potentially dependent on the mode of speciation and other contributing factors, including geographic setting and species traits. Several alternative proposals for the influence of range size on speciation rate have also been made (e.g. negative or a unimodal relationship with speciation). To examine Darwin’s proposal, we use a range of phylogenetic comparative methods, focusing on a large Australasian bird clade, the honeyeaters (Aves: Meliphagidae).

**Results:**

We consider the influence of range size, shape, and position (latitudinal and longitudinal midpoints, island or continental species), and consider two traits known to influence range size: dispersal ability and body size. Applying several analytical approaches, including phylogenetic Bayesian path analysis, spatiophylogenetic models, and state-dependent speciation and extinction models, we find support for both the positive relationship between range size and speciation rate and the influence of mode of speciation.

**Conclusions:**

Honeyeater speciation rate differs considerably between islands and the continental setting across the clade’s distribution, with range size contributing positively in the continental setting, while dispersal ability influences speciation regardless of setting. These outcomes support Darwin’s original proposal for a positive relationship between range size and speciation likelihood, while extending the evidence for the contribution of dispersal ability to speciation.

**Supplementary Information:**

The online version contains supplementary material available at 10.1186/s12862-022-02041-6.

## Background

How speciation rates vary among geographic regions and clades remains one of evolutionary biology’s most pivotal questions [1–3]. The influences on speciation of climate [4, 5], ecological opportunity [6, 7], latitudinal position [8, 9], traits [10], and geographic range size [11, 12] have been widely investigated. Yet conflict remains between theoretical expectations and empirical data. Particularly, the role of geographic range has yet to be resolved. Theory suggests that geographic range size should have a positive influence on the likelihood of speciation [1, 13, 14], although the form of this relationship may be influenced by the mode of speciation [15, 16]. Species with larger range sizes are expected to have a higher chance of speciation because of the larger area in which geographic barriers may form [17, 18]. Larger ranges tend to be associated with larger population sizes and genetic variability, and are more robust to localised environmental disturbances, also lowering the chance of extinction [11, 19–21].

Empirical investigations have found varying evidence for both positive [11, 19] and negative [10, 12, 21, 22] relationships between range size and speciation rate. Some theoretical arguments suggest that the species with the largest ranges may have ranges large enough to engulf barriers [18, 23], reducing speciation. However, smaller ranges are associated with higher extinction risk, also reducing speciation probability [20, 24, 25]. Thus, a further proposal has been made that geographic range size and speciation likelihood should show a unimodal relationship, in which intermediate ranges have the highest rate of speciation [14, 18, 23, 26].

**Fig. 1:**
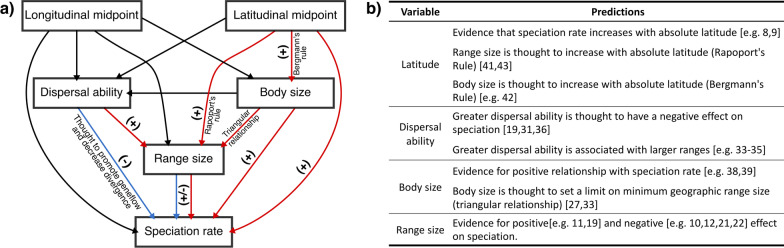
A conceptual diagram showing the range size speciation relationship and the theoretical influence of position (latitudinal and longitude), traits (body size and dispersal ability) on range size and speciation (**a**), with a table summarising the main hypotheses and predictions (**b**). Predicted positive and negative relationships are indicated with red and blue arrows respectively

Although current empirical support for a unimodal relationship is limited [27] and the idea has been described as unsatisfying [28], it remains appealing from a theoretical perspective because it also considers traits that influence the relationship between range size and speciation, such as dispersal ability and body size. Dispersal ability can either limit or promote gene flow, therefore affecting the probability of population divergence [22, 29–31], though this can depend on the mode of speciation [32]. Greater dispersal ability is associated with large range sizes [33–35], but is also thought to reduce speciation probability [19, 31, 36], complicating assessment of its effects on the relationship between geographic range size and speciation likelihood. Moreover, both range size and dispersal ability are also influenced by body size [33, 37], which in turn has an independent influence on speciation probability [38, 39] (Fig. 1).

Further complexity arises because the shape and geographic position of a species range can influence both range size and speciation likelihood. Shape can impact speciation because narrow or elongated ranges are more likely to give rise to dispersal events [40] and are thought to be prone to bisection by geographic barriers [13, 18], thereby increasing speciation probability, especially when geographic barriers are small [40]. Geographic position may act as a constraint on range size and is related to broader geographic gradients in both range size and body size. Acknowledging variation among taxa, range size and body size tend to decrease toward the tropics [41–43] (Fig. 1). Furthermore, species found in large biogeographic regions with a large spatial extent can have larger ranges than those found on small areas such as some islands [44, 45]. Moreover, island species derived from a continental relative are unlikely to recolonise the mainland [46] which can limit opportunity for range expansion in island species.

Any study seeking to understand the relationship between geographic range and speciation likelihood or rate has to take the interrelationship between traits and biogeographical constraints into account. The overall form of the relationship between geographic range size and speciation rate often emerges as weak, and often weakly negative [e.g. 10]. This could be the outcome of interactions between interrelated drivers of speciation, variation among taxa in traits such as body size and dispersal ability, and variation in biogeographic constraints between different major regions of the world (Fig. 1). One useful approach involves focusing on a large monophyletic group in order to understand the underlying causes and drivers of speciation at a range of scales [47–49].

We applied such an approach, using honeyeaters (Aves: Meliphagidae), to investigate the influence of geographic range size on speciation rate, taking into account the influences of range position, dispersal ability and body size. The honeyeaters are a large (192 species), diverse, monophyletic clade of passerine birds that are currently restricted to the east of Wallace’s line (except for one species found in Bali) and have diversified throughout Australasia in the past 25 million years [50–53]. Ranging from arid Australia to tropical Indonesia, the honeyeaters have diverged to fill a wide variety of niches and have considerable diversity in body size, dispersal ability and range size [50, 51]. The most common mode of speciation in birds is allopatric speciation in the broadest sense [17, 54]. This is evident in honeyeaters [55–57], and dispersal events throughout Indonesia and the South Pacific are thought to have played a prominent role [53, 58], indicative of peripatric speciation. Honeyeaters are well studied and described; distributions are known, and trait data are prevalent throughout the literature. Moreover, honeyeaters are distributed across both a large continent and many islands, and are monophyletic: ideal for examining the geographic range size idea in the expectation also of an influence of predominant modes of speciation. Here, we examine the hypotheses that: (i) the relationship between geographic range size and speciation rate is positive, and (ii) that the form of the relationship is likely to be influenced by the mode of speciation, while accounting for other factors that are likely to influence the geographic range size-speciation rate relationship (Fig. 1a).

## Results

### Phylogenetic reconstruction and range size

**Fig. 2:**
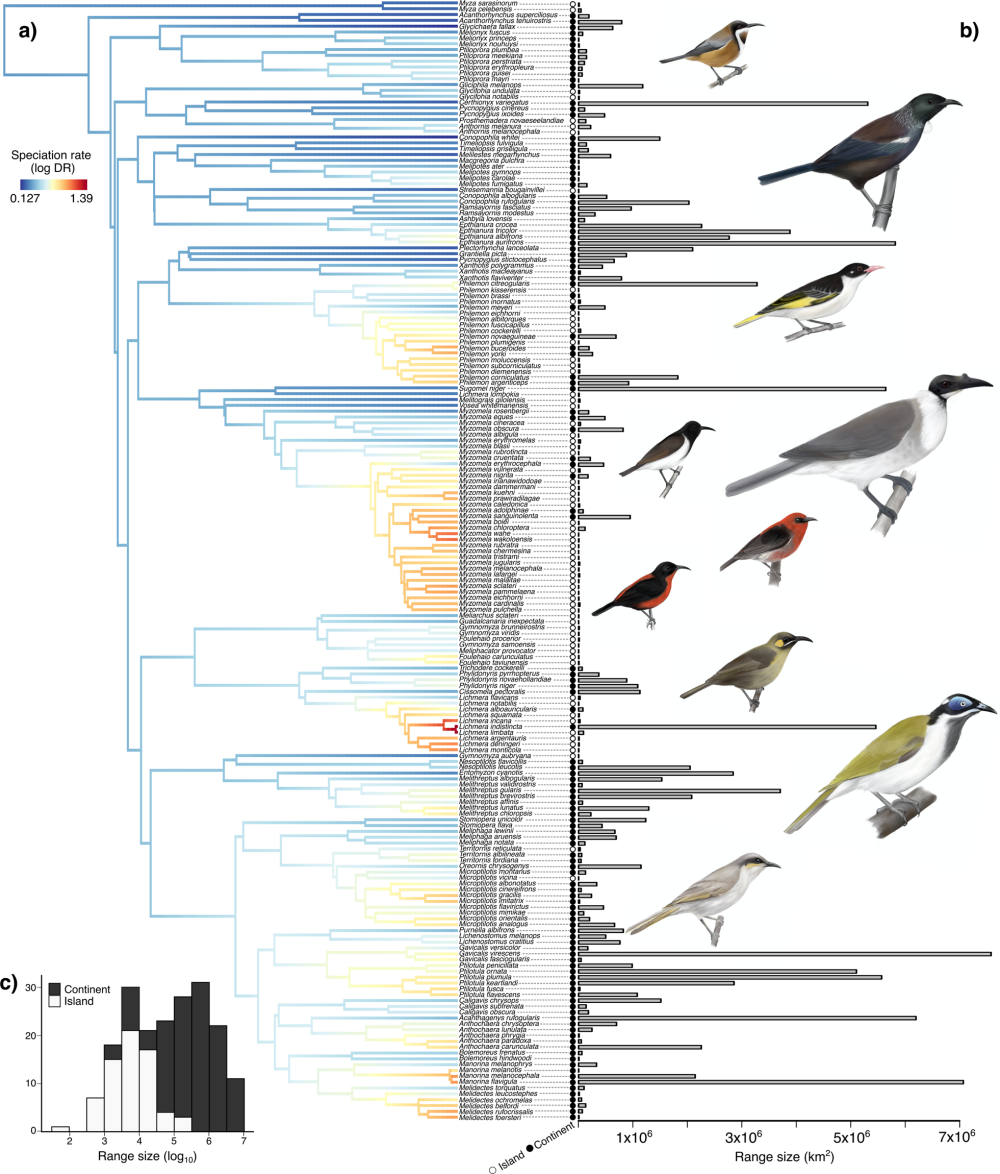
Time-calibrated phylogeny for the 192 honeyeater species (**a**). Coloured branches indicate speciation rate according to the DR statistic (log transformed). Dots indicate island and continental species, and the bars on the right indicate range sizes for each species (**b**). The distribution of range sizes (log transformed) for honeyeaters is displayed in the histogram (**c**) and bars are coloured according to island or continental species classification of species. Birds are illustrations by E.M.H. showing (from top to bottom) eastern spinebill (*Acanthorhynchus tenuirostris*), tūī (*Prosthemadera novaeseelandiae*), painted honeyeater (*Grantiella picta*), noisy friarbird *(Philemon corniculatus*), black honeyeater (*Sugomel niger*), scarlet honeyeater (*Myzomela sanguinolenta*), Rotuma myzomela (*Myzomela chermesina*), yellow-eared honeyeater *(Lichmera flavicans*), blue-faced honeyeater (*Entomyzon cyanotis*), and singing honeyeater (*Gavicalis virescens*)

We included ultra-conserved elements, mitochondrial and nuclear loci, alongside topological constraints from previous phylogenomic studies to generate a honeyeater phylogeny of 192 species (Fig. 2). The topology of genera within the tree is concordant with Andersen et al. [59] and relationships within the genus *Meliphaga* follow McCullough et al. [60]. In general, relationships between species within genera broadly follow previous reconstructions ([53]; see Additional file 1: Fig. S4 for a comparison).

The species with the smallest range size (42.4 km^2^) is the Rotuma myzomela (*Myzomela chermesina*), from Rotuma Island, Fiji. By contrast, the species with the largest range, the singing honeyeater (*Gavicalis virescens*), has a geographic range that spans nearly all of Australia (7,306,701 km^2^; mean honeyeater range size = 650,335.2 km^2^, median honeyeater range size = 78,121.45 km^2^).

### Phylogenetic regressions

**Fig. 3:**
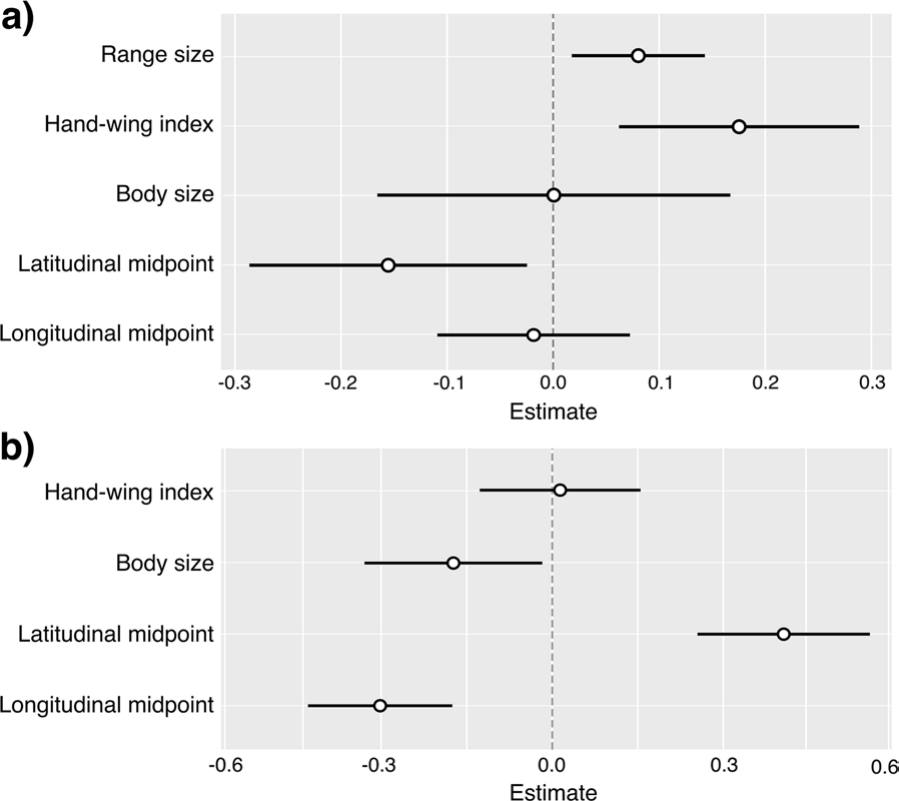
Results from phylogenetic generalised least squares regression models, testing the influence of variables on speciation rate (**a**) and traits and position on range size (**b**). Full model results are available in Additional file 2: Table S1. Bars indicate estimate and 95% confidence intervals. Note that for the PGLS results shown the predictor variables have been scaled so the effect sizes are comparable

Phylogenetic generalised least squares regressions revealed a positive relationship between range size and speciation rate (p = 0.01; Fig. 3a; Additional file 2: Table S1). In this model, speciation rate also increases with dispersal ability (p = 0.002) and declines with increasing latitudinal midpoint (p = 0.02; Fig. 3a). Species with less elongated or disjunct ranges are associated with higher rates of speciation (p = 0.02; Additional file 2: Table S1).

Geographic range size decreases with body size (p = 0.03; Fig. 3b). Range size and dispersal ability are unrelated, but dispersal ability and range shape show a positive relationship (p = 0.02; Additional file 2: Table S1). Range position is strongly correlated with range size, shape and dispersal ability (Additional file 1: Figs. S5–S11; Additional file 2: Table S1).

The geographic setting term, islands/continent, had a significant interaction with range size (p = 0.019), but not with dispersal ability (p = 0.124) or body size (p = 0.145; Additional file 2: Table S1). In the continental species only, higher rates of speciation are associated with larger ranges (p = < 0.0001; Additional file 1: Figs. S12–S15; Additional file 2: Table S1)

### Bayesian phylogenetic path analysis

Here, the only trait found to influence speciation rate is dispersal ability: species with greater dispersal abilities have higher rates of speciation. Range size is influenced by range position, with range size increasing with latitude and decreasing with longitude, and with continental range sizes larger than those on islands (Additional file 1: Fig. S16; Additional file 2: Tables S2, S3). Relationships between dispersal ability, range position, shape, and body size were in keeping with the phylogenetic regressions (Additional file 2: Table S1).

**Fig. 4:**
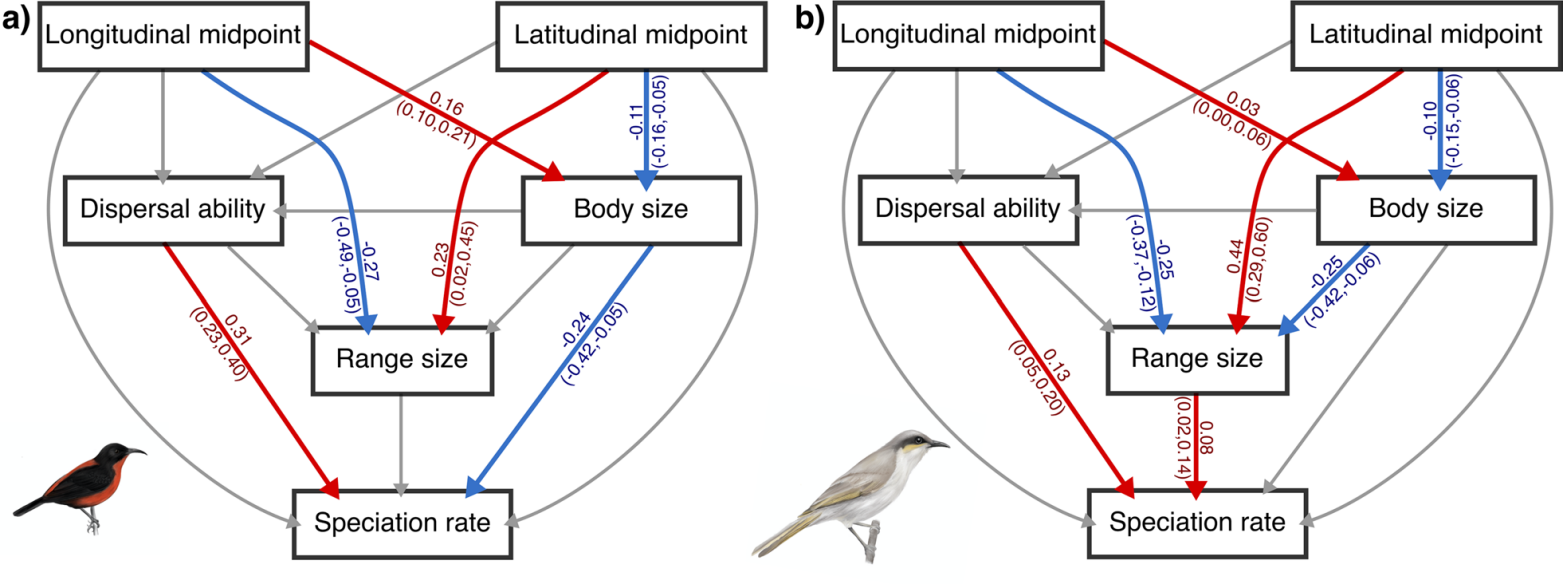
Results of the Bayesian structural equation path analysis on island species (**a**) and continental species (**b**). Positive, negative, and non-statistically significant relationships are indicated with red, blue, and grey arrows respectively, the estimate and 95% confidence intervals are displayed with the arrow. Full model results are available in Additional file 2: Tables S2–S5. Birds are illustrations by E.M.H., displayed are the honeyeater with the smallest range and an island species, the Rotuma myzomela (*Myzomela chermesina*) (**a**) and the honeyeater with the largest range and a continental species, the singing honeyeater (*Gavicalis virescens*) (**b**)

Due to the influence of islands on species range size, analysis was repeated separately on island and continental species. In island species speciation rate is related to high dispersal ability and small body size (Fig. 4a; Additional file 2: Tables S4, S5). By contrast, in the continental species, higher speciation rates are significantly associated with larger range sizes and greater dispersal ability (Fig. 4b; Additional file 2: Tables S4, S5). In both island and continental species, range size increases with latitude and decreases with longitude. Body size is also found to be influenced by position, decreasing with latitude and increasing with longitude (Fig. 4).

### Spatiophylogenetic model

**Fig. 5:**
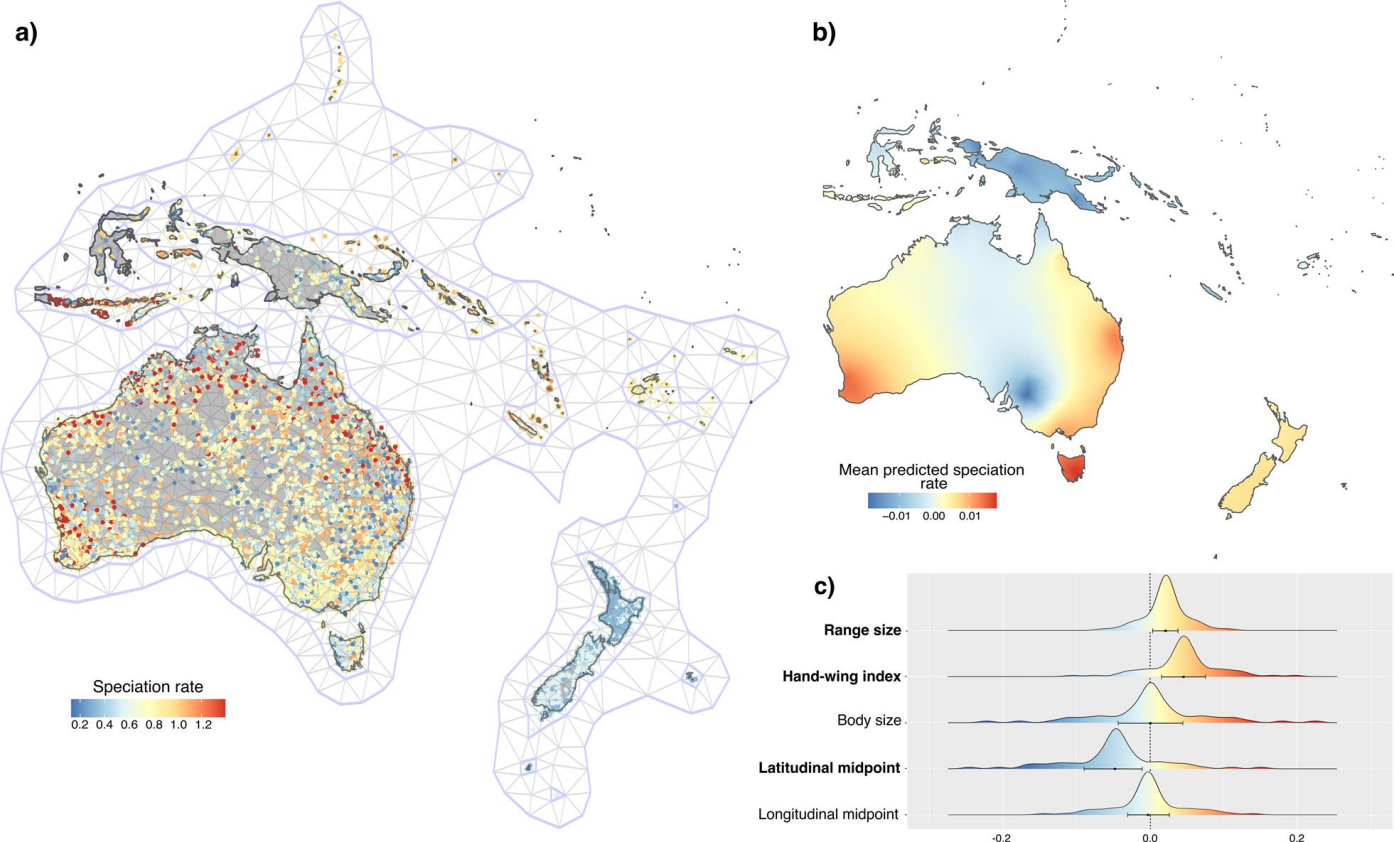
A summary of the spatiophylogenetic model results. **a** Shows the 254,886 honeyeater point occurrence records which have been coloured according to speciation rate (DR statistic), overlayed is the spatial mesh consisting of 877 vertices which was constructed over the point occurrence records and averaged across the spatial random fields of each occurrence record for each species. **b** Shows the mean predicted speciation rate extracted from the final spatiophylogenetic model. **c** Is a density plot of the marginals of the fixed effects in the model, with the mean and 95% credible intervals. Full model results are available in Additional file 2: Table S6. Predictor variables which were identified to have significant effects are highlighted in bold

The spatiophylogenetic model revealed considerable spatial and phylogenetic structure to the variation in speciation rate (Fig. 5). Speciation rates are variable across both the continental and island settings, as the previous analyses indicated, and range size retains a positive relationship with speciation rate. So does dispersal ability, and the negative relationship with latitudinal midpoint, indicating higher rates in the tropics, is also retained (Fig. 5c; Additional file 2: Table S6).

### State-dependent speciation and extinction analyses

The state-dependent speciation and extinction models verified the influence of range size on speciation rate, though at first the outcomes seem to contradict the previous model outcomes. The FiSSE model closest to significance for range size identified small ranges less than 100,000 km^2^ as having higher rates of speciation than range sizes larger than this value (λ_small range_ = 5.96, λ_large range_ = 4.61, p = 0.058; Additional file 2: Tables S7, S8). Four HiSSE models, with Akaike weights 0.66, 0.067, 0.056, and 0.051, respectively, occupy the bulk of the model weight (*w*_*i*_ = 0.834), with the remainder of the weight associated with a CID-4 model (*w*_*i*_ = 0.143; Additional file 2: Tables S9, S10). Importantly, however, the FiSSE models also confirmed that island species have higher speciation rates than their continental counterparts (λ_islands_ = 6.47, λ_continents_ = 4.68, p = 0.049; Additional file 2: Tables S7, S8). This was supported by the HiSSE modelling, where the best HiSSE model (*w*_*i*_ = 0.952) outperformed the four CID models which were included as null models (All have *w*_*i*_ ≤ 0.001; Additional file 2: Tables S9, S10). Differences in the range size distributions of the species on islands and the continent provides the explanation (Fig. 2c). When classified on a binary basis, ~ 96% (65 of 68) of the island-dwelling species have range sizes less than 100,000 km^2^ in extent, whereas ~ 72% (89 of 124) of the continental species have ranges larger than this threshold.

Such a finding is in keeping with the phylogenetic regressions, spatiophylogenetic modelling and the structural equation models demonstrating the importance of separating the continental from the island settings. If the differences between island and continental settings are significant, then a state-dependent speciation and extinction analysis differentiating large and small range-sized species from those with intermediate-sized ranges should reveal an effect with the former having the higher values. A FiSSE model using the quartile range to make such an adjudication (i.e. grouping the lower and upper quartiles) is indicative that this may be the case (FiSSE: λ_intermediate range_ = 4.92, λ_small + large range_ = 5.71, p = 0.081; Additional file 2: Tables S7, S8). Perhaps more noteworthy, the HiSSE model adopting the same classification outperforms (*w*_*i*_ = 0.688) the CID models (best CID model *w*_*i*_ = 0.107; Additional file 2: Tables S9, S10).

## Discussion

Theory suggests that larger ranges should have the highest speciation rates, with these relationships being potentially modified both by the mode of speciation and by other contributing factors and species traits [18, 34, 36, 37]. The latter, such as dispersal ability and range spatial position [12, 35, 40], may have independent effects on both range size and the likelihood of speciation [30, 36]. When this complexity is taken into account by applying several analytical approaches, the positive relationship between range size and speciation rate is supported, and a significant interaction found between range size and islands indicates that the patterns vary depending on geographic setting, which likely reflects mode of speciation. Specifically, honeyeater speciation rate differs considerably between islands and the continental setting across the clade’s distribution, with dispersal ability influencing speciation rate regardless of setting and range size contributing positively in the continental setting. The higher rates in the tropics are, in part, a reflection of the differential distribution of the continental and island settings. Therefore, in the Meliphagidae it is clear that dispersal associated speciation across islands and range size related allopatric speciation in the continental setting have both contributed to diversification. Moreover, we have been unable to reject the two hypotheses we sought to test, lending support both to theory and to previous findings for the honeyeaters.

In the case of the first hypothesis (a positive relationship between range size and speciation likelihood) [1, 17, 18], our results support the idea that, where vicariant speciation is likely to be the dominant mode of allopatric speciation, a positive relationship with range size is to be expected. Darwin [1] originally proposed that larger ranges would have a greater chance of allopatric speciation events. These ideas have persisted [13, 23] and empirical studies have found positive relationships between range size and speciation [11, 19]. By contrast, our results do not support the hypothesis of a largely negative or unimodal relationship between range size and speciation [10, 27].

Among Australian continental vertebrates, including birds, patterns of vicariant speciation associated with changing climates in particular, but also landform evolution, have played a major role in species diversification [61–63]. Diversification patterns and their outcomes are similar among mammals, amphibians, and birds [64] and our findings for continental honeyeaters are congruent with these patterns (Fig. 5). Examples include high speciation rates of honeyeaters on the east coast, with a hotspot around the Atherton Tableland in eastern Queensland (Fig. 5). Additionally, environmental heterogeneity and aridification of Australia has resulted in increased diversification of arid-zone clades in vertebrates [65]. The increasing aridification of Australia has also resulted in shrinking mesic biomes, restricting species ranges, driving extinction and vicariant speciation. This environmental heterogeneity and the shifting of biomes has had widespread effects and facilitated allopatric speciation in mammals [66], reptiles [67], fish [68], and amphibians [69] across Australia. Although we have not investigated the specific historical environmental drivers of speciation, our results appear to be consistent with these patterns (Fig. 5) and previous investigations of barriers influencing honeyeaters [56, 57].

Nonetheless, specific identification of vicariant speciation as important for the honeyeaters is new. Honeyeaters are well established as good dispersers and have longer and more projected wings compared to their meliphagoid relatives [29, 70]. Vicariance events have been suggested to have acted in this group [53, 71], but to date, evidence for speciation in honeyeaters has predominantly supported the role of dispersal events throughout Indonesia and the South Pacific [53, 55, 72], associated with their nectarivorous lifestyle [35, 70]. Although we confirm the results of previous honeyeater studies which have suggested that dispersal has been key to honeyeater speciation especially in island species [53, 55], we find that vicariant patterns associated with a large range size mechanism in the continental setting is also important. Differences between the continental and island settings are supported by all the analytical approaches, including the state-dependent diversification models. In both settings, dispersal is an important contributor to diversification rate, but in the continental setting, range size contributes too.

The finding of smaller ranges associated with higher rates of speciation is consistent with previous studies that have investigated range size and speciation in birds [10, 12]. This result is, however, at least partially driven by island endemism. Higher rates of speciation are evident in species from the genera *Philemon*, *Myzomela*, and *Lichmera*, all of which include a greater proportion of island than continental species (Fig. 2). Such patterns of island speciation became apparent when plotting occurrence records coloured according to speciation rate (Fig. 5a), and small islands such as those throughout the Lesser Sunda Islands and the Banda Arc are still visually associated with higher rates of speciation in the predicted speciation rate map from the spatiophylogenetic analysis (Fig. 5b). This finding of islands as speciation hotspots is not surprising given that islands frequently play key roles in avian speciation [e.g. 12, 73, 74]. The island setting is clearly characterised by dispersal-associated speciation. High dispersal ability typically improves the ability of species to reach oceanic islands, increasing rates of diversification, which frequently play key roles in avian speciation [16], as found for the honeyeaters [53, 55, 72].

Latitudinal gradients in speciation rates have been well investigated and the subject of much theoretical discussion [75]. Most recently, empirical studies have often uncovered patterns in which speciation rates decrease towards the tropics [8, 9]. Although we find evidence for a latitudinal gradient in honeyeater speciation rates, suggesting higher rates in the tropics, the outcome appears to have more to do with the island versus continental setting than a tropical versus extratropical setting, though obviously the two cannot entirely be distinguished. The outcome is wholly in keeping with the geographic complexity that may be expected from the differences between clades and environmental settings and their interactions [75]. It also highlights the benefits of a focus on such settings and specific taxa for distinguishing the underlying likely influences on macroecological and macroevolutionary patterns [47].

Inferences of modes of speciation from current ranges should be cautious, as current ranges may not always be representative of past evolutionary history. However, simulations have demonstrated that even if species ranges have changed over time, speciation history is often detectable from current distributions [76].

## Conclusions

Our results indicate that the relationship between geographic range size and speciation rate is positive and is influenced by mode of speciation. However, relationships between other factors are likely to influence the geographic range size-speciation rate relationship, including dispersal ability, and accounting for covariance due to shared biogeographic occupancy is also critical. Our ability to understand the proximal basis of speciation rate variation across the Tree of Life can be greatly aided by elucidating these complex multi-trait relationships.

## Methods

### Phylogenetic inference

We used recent phylogenomic analyses [59, 60] in conjunction with traditional nuclear and mitochondrial markers [53] to construct a comprehensive phylogeny of honeyeaters. We followed the IOC world bird list (version 10.2; [77]), which recognises 191 species of honeyeater and follows recent taxonomic revisions suggested by genomic work [60, 69], including the recently described *Myzomela* species [78–80]. We included *Myzomela obscura rubrotincta* as a distinct species, *Myzomela rubrotincta*, due to previous work suggesting that this represents a distinct species based on morphology and vocalisations [81] and molecular work finding this species has been more closely related to *M. cruentata* than to *M. obscura* [53].

All available DNA sequences for Meliphagidae species were downloaded from GenBank. This included sequences for eight nuclear genes (Fib-5, GAPDH, RAG-1, RAG-2, FIB-B17, c-mos, BDNF and TGBF2) and five mitochondrial genes (12 S, cyt-b, COI, ND2 and ND3). The coverage for each of these genes varied from 3 species to 187 species (Additional file 2: Table S12). We included the following nine taxa as outgroups; *Gerygone chloronota*, *Gerygone chrysogaster*, *Acanthiza apicalis*, *Acanthiza chrysorrhoa*, *Dasyornis broadbenti*, *Pardalotus striatus*, *Pardalotus punctatus*, *Sericornis frontalis* and *Sericornis perspicillatus* (Additional file 2: Table S12). Sequences were aligned for each loci using MUSCLE [82]. From here, individual gene trees were generated for each of the nuclear and mitochondrial genes using IQ-TREE [83]. This was implemented in the IQ-TREE web server with an automatic substitution model in which IQ-TREE determines the best-fit substitution model for the data [83, 84].

For the phylogenomic data we used information from two recent phylogenomic studies based on ultraconserved elements (UCE); one resolved genus-level relationships of honeyeaters [59] and the second resolved some species-level relationships within the genus *Meliphaga* [60]. Unfortunately, sequence data for ultraconserved elements were not publicly available from the genus-level study [59]. However to include the consideration of honeyeater genus-level relationships resolved from this study we extracted the topology of the ASTRAL tree presented as Fig. 3 by Andersen et al. [59] to use as a constraint tree. UCEs were downloaded for 12 species of honeyeater ([60]; BioProject PRJNA509981; see Additional file 2: Table S12 for UCE accession numbers and the species included in the constraint tree). Ultraconserved elements were clustered based on locus, aligned using MAFFT [85], and trees for each locus were generated with IQ-TREE [83] using a mixed GTR model. A total of 4676 gene trees was generated for ultraconserved elements.

The 13 gene trees and 4676 UCE trees were used as input for ASTRAL (Accurate Species TRee ALgorithm; [86]), alongside the genus-level topology of honeyeaters from Andersen et al. [59] which was used as a constraint tree. ASTRAL provides a statistically consistent estimation of the true species tree from unrooted gene trees, under the multi-species coalescent model [86]. The resulting ASTRAL topology tree (Additional file 1: Fig. S1) was then calibrated using a penalized likelihood approach implemented in the program treePL [87, 88] (see Additional file 3 for further details on an additional calibration method).

For the penalised likelihood approach, we first needed to estimate branch lengths of the tree in substitutions per site. To do so we used RAxML-NG [89]. Here, we used the ASTRAL topology tree and the concatenated alignment of the 13 nuclear and mitochondrial loci, to optimize branch lengths and free model parameters on a fixed topology. The concatenated alignment was partitioned by loci and codon position, and we assigned a separate GTR + I model of rate heterogeneity to each locus. The resulting tree was then calibrated using penalized likelihood implemented in the program treePL [87, 88]. TreePL explicitly allows for rate variation across branches but penalises rate differences after cross-validating initial analyses. Unfortunately, fossil coverage of the honeyeaters is poor; only one honeyeater fossil is known [90]. Since the primary hypotheses being examined in this study do not require accurate dating of the tree, this was not done, and the root of the phylogeny was fixed to one.

Finally, to have a fully sampled phylogenetic tree for all honeyeater species the two missing species, *Melipotes carolae* and *Myzomela dammermani* were added into the phylogeny using TACT [91]. The placement of these species was based on taxonomic suggestions from Birds of the World available through the Cornell Lab of Ornithology [92].

### Range size and phenotypic trait variables

We obtained range data for all honeyeater species from BirdLife International [93]. Range size based on interpolated range polygons, such as the BirdLife International range maps, tends to over-estimate the true extent of occurrence, which may be a limitation to this study, but which is an unbiased one relative to the hypotheses being tested. Following previous studies which have also used these maps [12, 35], we only considered extant resident and breeding ranges to avoid introducing bias between migratory versus non migratory species and areas where species have been introduced. We used Quantum Geographic Information System (QGIS; [94]) to extract information relating to range size, shape and position. Geographic range size was extracted (km^2^). Range shape was quantified as the total range area divided by the total perimeter for each species. Using this index, species with smaller values (smaller area and a larger perimeter) have more elongated or disjunct ranges [12]. Here, we also considered latitudinal and longitudinal extent as important aspects of shape, which are thought to be related to probability of range bisection [13, 18]. Extent was calculated by generating a bounding box around each species range and extracting the length and width of the range extent in kilometres. We characterised range position using two approaches; centroids were extracted from polygons for each species range, which were calculated in QGIS and are influenced by the shape of a species’ range, and we also extracted latitudinal and longitudinal midpoints from the range extent. Both approaches are commonly used and despite being slightly different, these two measures were highly correlated (Additional file 1: Fig. S5) and so midpoints were only considered for analysis.

Here, we also noted whether species were classified as island endemic species or continental species. Species whose ranges overlapped with Australia, Tasmania and New Guinea were considered continental species (n = 124) and all other species as island species (n = 68). This classification takes into consideration the historical biogeography of the region. Both Tasmania and New Guinea sit on continental crust and have been repeatedly connected to Australia via the Sahul Shelf throughout the Pleistocene [95], which has enabled considerable biotic interchange and gene flow in the Australo-Papuan region.

To examine factors that may contribute to or influence the geographic range size speciation relationship we included measures of body size and dispersal ability. Measures of body size, using mass in grams, were extracted from the literature [96]. We used a similar approach to previous datasets [35], where for species without direct measurements of body mass we substituted missing values using genus averages, considering the same species list used for the phylogeny ([77]; Additional file 2: Table S11). Dispersal ability was assessed by using the hand-wing index (HWI), an index of wing shape commonly used as an indicator of flight performance and dispersal capability [29, 30]. HWI was calculated as Kipp’s distance (the distance between the tip of the first secondary feather to the tip of the longest primary feather, measured on the folded wing) divided by wing length, then multiplied by 100. Measurements of wing length and Kipp’s distance were extracted from Marki et al. [70], these measurements were averaged for each species and hand-wing index was calculated. Additional values of HWI were then substituted from Sheard et al. [35]. From here, HWI values were missing for five species of honeyeater (*Melipotes carolae, Myzomela prawiradilagae*, *Myzomela irianawidodoae*, *Myzomela wahe*, and *Microptilotis imitatrix*) and these were substituted in using phylogenetic imputation implemented in R [97] using the package ‘phytools’ [98] (Additional file 2: Table S11).

### Speciation rate estimates

To obtain a continuous estimate of speciation rate for use in phylogenetic regressions, phylogenetic path analysis and the spatiophylogenetic model, we used the diversification rate (DR) statistic. This was calculated in R [97] using the *DR_statistic* function from the ‘fisse’ R package [99]. The DR statistic was selected estimates tip-specific rates of diversification without a formal parametric model, has been shown to provide a better estimate of speciation than net diversification, and performs as well as other commonly used statistics [100, 101]. We additionally used hidden-state dependent speciation and extinction models as a complementary approach, which estimate their own rates of speciation [102].

### Phylogenetic regressions

To test the relationship between range size and speciation, while accounting for other factors that are likely to influence this relationship, measures of range size, shape, and position for each species were treated as traits and used in phylogenetic generalised least-squares regressions (PGLS) alongside measures of body size and dispersal ability [103]. Continuous variables were log transformed to improve normality and absolute values of latitude and longitude were used. PGLS regression tests were run using the ‘ape’ (v.5.3 [104]) and ‘nlme’ [105] packages in R [97]. An estimated lambda parameter was used to control for the amount of phylogenetic effect in the model residuals [106, 107]. To examine whether relationships differed between islands and continents, we tested an interaction term in our analysis. We reasoned that if the interaction term is significant, examination of our second hypothesis, that the form of the geographic range size-speciation relationship is influenced by mode of speciation, could be furthered by phylogenetic regression undertaken separately for the island and continental species. Previous studies of other bird clades have employed separate examinations of island and mainland species [12], and we adopted this rationale (additional split regression analyses, including those with nonsignificant interaction terms, are present in Additional file 2: Table S1).

### Phylogenetic bayesian structural equation models

To further uncover the relationship between range size and speciation, we implemented a Bayesian version of phylogenetic path analysis [108]. This analysis was undertaken in R [97] using ‘brms’ [109], for Bayesian multilevel models using the probabilistic programming language STAN [110, 111]. Unlike simple phylogenetic regressions, the use of structural equation models enables us to test the multiple interactions between all variables and traits in a single model, an ideal approach to determine the relationship between range size and speciation while accounting for other factors that are likely to influence this relationship (Fig. 1a). Specific model details can be found in Additional file 3. Path analyses were performed on all species, and separately on island and continental species following the rationale above, enabling us to test our second hypothesis that the form of the geographic range size-speciation relationship is influenced by mode of speciation.

### Spatiophylogenetic modelling

Spatiophylogenetic modelling [112] was then used to estimate the spatial and phylogenetic structure in speciation rate, and to examine the contributions of range size, dispersal ability, body size and range position to variation in speciation rate (DR statistic). To model the spatial distributions of each species, we downloaded available occurrence records for Meliphagidae from eBird via GBIF [113, 114] (see Additional file 2: Table S13 and Additional file 3 for further details), our analysis included a total of 254,886 honeyeater occurrence records.

The spatiophylogenetic model was implemented in R using the package “INLA” which uses an integrated nested Laplace approximation to estimate the joint posterior distribution of model parameters [115, 116]. INLA is an approach for latent Gaussian Markov random field models, providing a significantly faster alternative to Markov chain Monte Carlo [116, 117]. We tested the influence of range size, position (latitudinal and longitudinal midpoints), dispersal ability and body size on speciation rate and included both a phylogenetic random effect and spatial random effect in the model. For the phylogenetic effect, INLA requires a phylogenetic precision matrix which is the inverse of a phylogenetic covariance matrix. Prior to inverting, the phylogenetic covariance matrix was standardised by dividing by its determinant raised to the power of 1/N_species_ [112]. To model the spatial effect across the entire landscape, we used a spatial mesh, which was constructed over the point occurrence records and averaged across the spatial random fields of each occurrence record for each species. This approach integrates a spatial random field across each species’ distribution, so each species contributes a single datapoint to the likelihood, reducing any bias that could arise from biased data in occurrence records such as clustered data due to differential sampling effort, or when the number of occurrence records varies between common and rare species (see [112] for further model explanation). We used a mesh consisting of 877 vertices and used default priors for both the phylogenetic and spatial effects. The mesh selected provided a suitable coverage of honeyeater distributions and allowed reasonable computational times. Prior to analysis, all predictor variables were standardised by subtracting the mean and dividing by the standard deviation. The significance of effects in the models were based on whether the 95% credible intervals of the effect size overlapped with zero.

### State-dependent speciation and extinction analyses

To verify which factors influence speciation rates in honeyeaters, we applied two methods of state-dependent speciation and extinction analyses: FiSSE (Fast, intuitive State-dependent Speciation-Extinction analysis [99]) and HiSSE (Hidden State-dependent Speciation and Extinction [102]). We first used the R package “fisse” [99] to explore trait-dependent speciation. We then used HiSSE [102] to verify the FiSSE outcome. HiSSE represents an improvement on previous models [118, 119] as it not only models the influence of a binary character on observed speciation rates, but also models an unobserved hidden character that could potentially impact species diversification rates.

The FiSSE analyses explored a range of thresholds to determine whether an association might exist between each trait and diversification rates. Thresholds were classified based on frequency distributions of the data, and the median, 25% and 75% quartiles (Additional file 2: Tables S8, S9). HiSSE models were then investigated where for any given trait, the FiSSE analyses revealed significant, or near significant (p < 0.07) outcomes (Additional file 2: Tables S8, S9). Using the R package “hisse” [102], twenty different models were fitted. Sixteen of these are HiSSE approaches that model the observed states (0 and 1A), but also assume a hidden state with each of the observed states (0B and 1B). These models estimate speciation and extinction for each of the four states and have transition rates between each of them, with each of the models representing various constraints on the transition rate matrix [102]. The remaining four are null models, corresponding to various forms of character independent diversification (CID). Two CID-2 models are equivalent in complexity to a binary SSE model, and two CID-4 models are equivalent in complexity to the full HiSSE model [102]. As our tree (n = 192) is below the threshold (n = 800) necessary to recover unbiased individual transition rates [102] we focus our attention on the AIC and relative model weights of HiSSE and CID models.

## Supplementary Information

**Additional file 1: Figure S1.** Honeyeater topology tree estimated with ASTRAL, generated from 4676 UCE trees of 12 honeyeater species, 13 mitochondrial and nuclear gene trees (coverage varying from 3 species to 187 species), alongside a constraint tree extracted from Andersen et al. [59] that resolves genus-level relationships of honeyeaters. **Figure S2.** Dated phylogeny of honeyeaters, generated in MCMCtree using the topology tree from ASTRAL alongside the fully partitioned and concatenated dataset of all mitochondrial and nuclear genes, with the root of the phylogeny fixed to one. Node labels show the estimated age. Error bars represent 95% highest posterior densities for estimated node age. **Figure S3.** Dated phylogeny of honeyeaters, generated in TreePL using the topology tree from ASTRAL alongside the fully partitioned and concatenated dataset of all mitochondrial and nuclear genes, with the root of the phylogeny fixed to one. Node labels show the estimated age. **Figure S4.** Tanglegram comparing the topology of the phylogeny generated in this study (left) with that obtained from previous studies (right; [53]). Colours indicate similarities and the black lines indicate topological differences, with the lines matching the same species between the two trees. Note the Marki et al. [53] phylogeny contains 186 honeyeater species and whereas the tree from this studycontains 192 species, but here it has been trimmed to the same 186 species. **Figure S5.** Correlation plot indicating relationships between all traits and range size variables included in this study. **Figure S6.** Biplots showing the relationship between traits and variables included in this study. Trendlines and p-values are generated from PGLS regressions, using the tree from the TreePL calibration method. **Figure S7.** Biplots showing the relationship between traits and variables included in this study. Trendlines and p-values are generated from PGLS regressions, using the tree from the TreePL calibration method. **Figure S8.** Biplots showing the relationship between traits and variables included in this study. Trendlines and p-values are generated from PGLS regressions, using the tree from the TreePL calibration method. **Figure S9.** Biplots showing the relationship between traits and variables included in this study. Trendlines and p-values are generated from PGLS regressions, using the tree from the MCMCtree calibration method. **Figure S10.** Biplots showing the relationship between traits and variables included in this study. Trendlines and p-values are generated from PGLS regressions, using the tree from the MCMCtree calibration method. **Figure S11.** Biplots showing the relationship between traits and variables included in this study. Trendlines and p-values are generated from PGLS regressions, using the tree from the MCMCtree calibration method. **Figure S12.** Results from PGLS regressions showing the relationship between traits and variables included in this study, subset into island species (n = 68; top row—blue panels) and continental species (n = 124; bottom row—red panels). Trendlines and p-values are generated from PGLS regressions, using the tree from the TreePL calibration method. **Figure S13.** Results from PGLS regressions showing the relationship between traits and variables included in this study, subset into island species (n = 68; top row—blue panels) and continental species (n = 124; bottom row—red panels). Trendlines and p-values are generated from PGLS regressions, using the tree from the TreePL calibration method. **Figure S14.** Results from PGLS regressions showing the relationship between traits and variables included in this study, subsetinto island species (n = 68; top row—blue panels) and continental species (n = 124; bottom row—red panels). Trendlines and p-values are generated from PGLS regressions, using the tree from the MCMCtree calibration method. **Figure S15.** Results from PGLS regressions showing the relationship between traits and variables included in this study, subset into island species (n = 68; top row—blue panels) and continental species (n = 124; bottom row—red panels). Trendlines and p-values are generated from PGLS regressions, using the tree from the MCMCtree calibration method. **Figure S16.** Full phylogenetic Bayesian structural equation model testing the influence of range position (latitudinal and longitudinal midpoints) and islands, on body size and dispersal ability, as well as the interaction between body size and dispersal, and the influence of all these traits on range size, range shape, and ultimately speciation. Model results: Blue arrows indicate a significant negative relationship; Red arrows indicate a significant positive relationship. Grey arrows indicate no significant relationship.

**Additional file 2: Table S1.** PGLS results (both TreePLtree and MCMCtree tree). **Table S2. **SEM results TreePL tree. **Table S3. **SEM results MCMCtree tree. **Table S4. **SEM results island vs. continental species TreePL tree. **Table S5.** SEM results island vs continental species MCMCtree tree. **Table S6.** INLA model results (bothTreePL tree and MCMCtree tree). **TableS7.** FiSSE results (TreePL tree). **TableS8.** FiSSE results (MCMCtree tree). **Table S9.** HiSSE results (TreePL tree). **Table S10.** HiSSE results (MCMCtree tree). **Table S11.** Honeyeater trait dataset. **Table S12.** Accession table, contianing the GenBank accession numbers and list of species and outgroups included in this study. **Table S13.** Simulated occurrence data for missing honeyeater species.

**Additional file 3.** Supplementary text file.

## Data Availability

Additional material is available from Figshare at 10.26180/19375091.
