# Supplementary material for: Geographic range size and speciation in honeyeaters
Source: BMC Ecol Evol. 2022 Jun 29;22:86. doi: 10.1186/s12862-022-02041-6 (PMC9245323; doi:10.1186/s12862-022-02041-6)
Supplement: Supplementary file 1 — Additional file 1: Figure S1. Honeyeater topology tree estimated with ASTRAL, generated from 4676 UCE trees of 12 honeyeater species, 13 mitochondrial and nuclear gene trees (coverage varying from 3 species to 187 species), alongside a constraint tree extracted from Andersen et al. [59] that resolves genus-level relationships of honeyeaters. Figure S2. Dated phylogeny of honeyeaters, generated in MCMCtree using the topology tree from ASTRAL alongside the fully partitioned and concatenated dataset of all mitochondrial and nuclear genes, with the root of the phylogeny fixed to one. Node labels show the estimated age. Error bars represent 95% highest posterior densities for estimated node age. Figure S3. Dated phylogeny of honeyeaters, generated in TreePL using the topology tree from ASTRAL alongside the fully partitioned and concatenated dataset of all mitochondrial and nuclear genes, with the root of the phylogeny fixed to one. Node labels show the estimated age. Figure S4. Tanglegram comparing the topology of the phylogeny generated in this study (left) with that obtained from previous studies (right; [53]). Colours indicate similarities and the black lines indicate topological differences, with the lines matching the same species between the two trees. Note the Marki et al. [53] phylogeny contains 186 honeyeater species and whereas the tree from this studycontains 192 species, but here it has been trimmed to the same 186 species. Figure S5. Correlation plot indicating relationships between all traits and range size variables included in this study. Figure S6. Biplots showing the relationship between traits and variables included in this study. Trendlines and p-values are generated from PGLS regressions, using the tree from the TreePL calibration method. Figure S7. Biplots showing the relationship between traits and variables included in this study. Trendlines and p-values are generated from PGLS regressions, using the tree from the TreePL calibration method. Figure [file 12862_2022_2041_MOESM1_ESM.pdf]

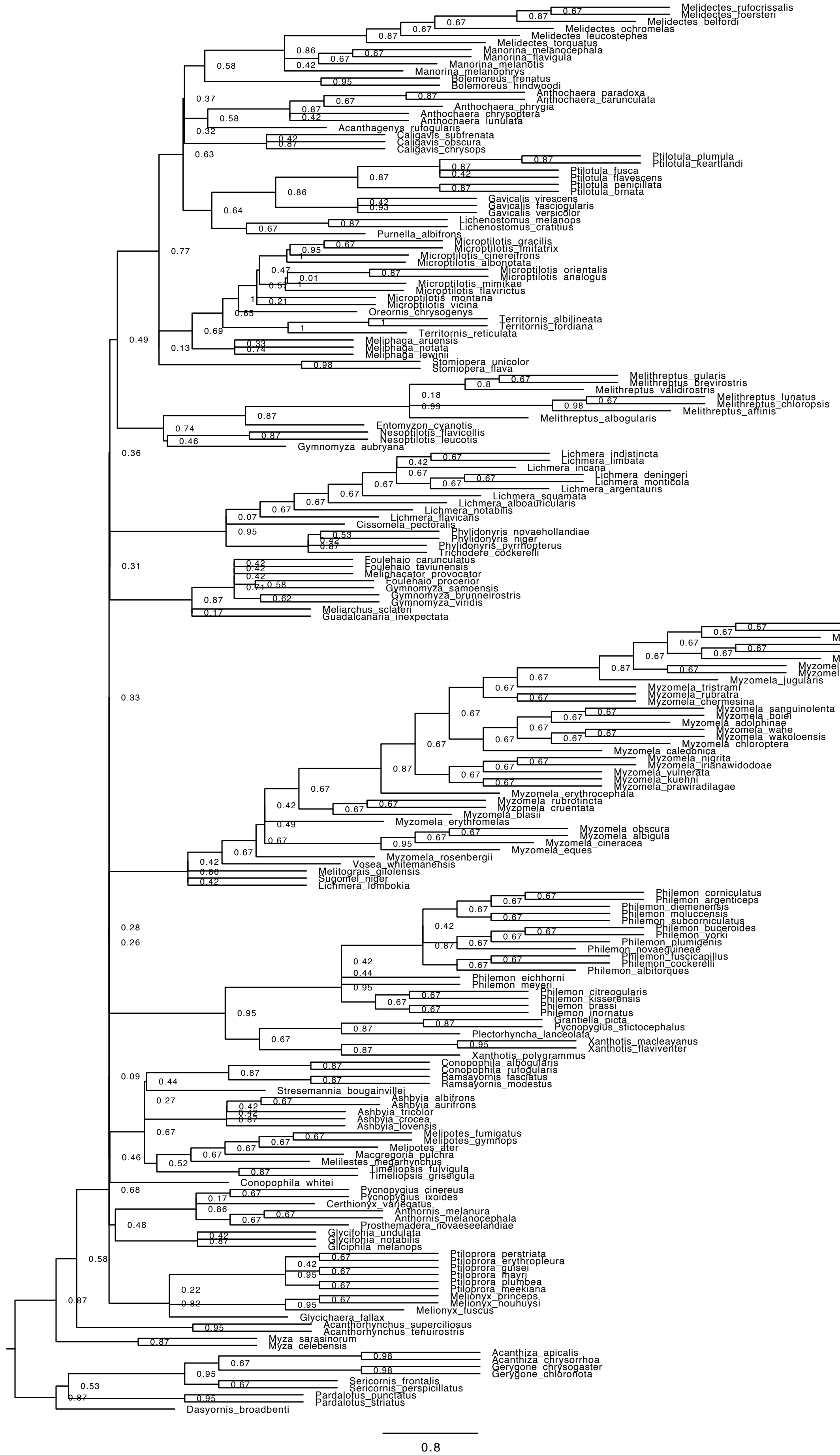

Figure S1. Honeyeater topology tree estimated with ASTRAL, generated from 4676 UCE trees of 12 honeyeater species, 13 mitochondrial and nuclear gene trees (coverage varying from 3 species to 187 species), alongside a constraint tree extracted from Andersen *et al.* (2019) that resolves genus-level relationships of honeyeaters.

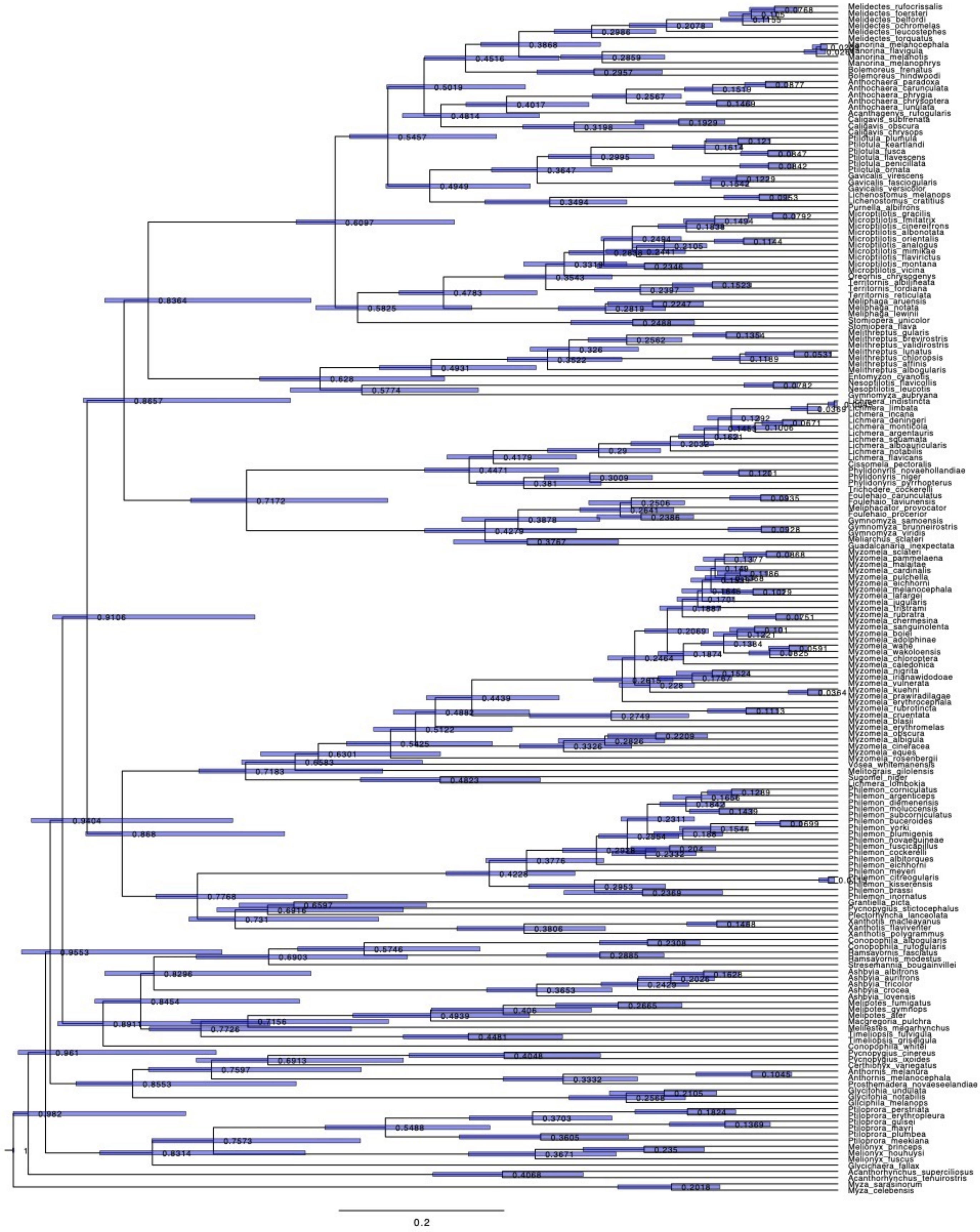

Figure S2. Dated phylogeny of honeyeaters, generated in MCMCtree using the topology tree from ASTRAL alongside the fully partitioned and concatenated dataset of all mitochondrial and nuclear genes, with the root of the phylogeny fixed to one. Node labels show the estimated age. Error bards represent 95% highest posterior densities for estimated node age.

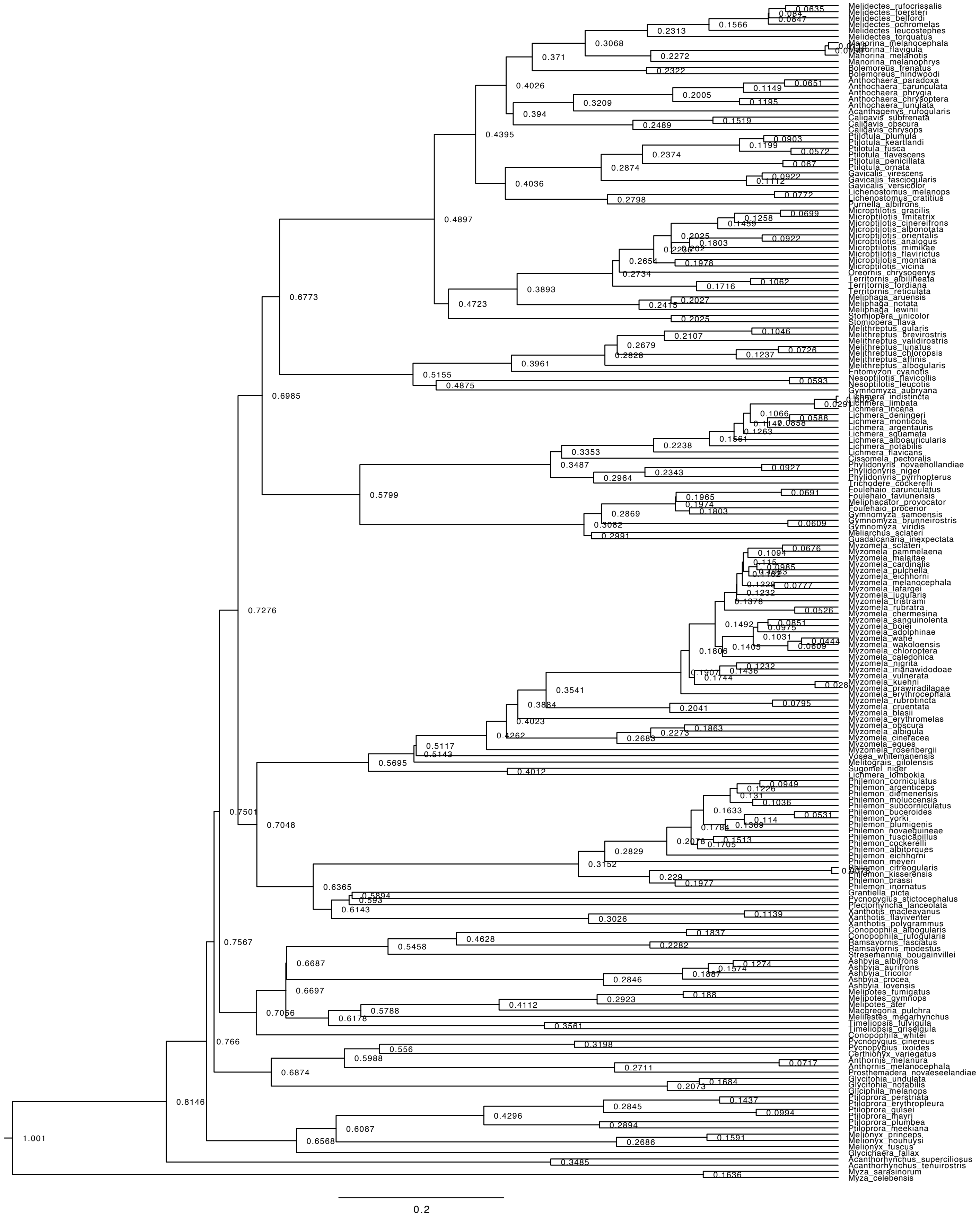

Figure S3. Dated phylogeny of honeyeaters, generated in TreePL using the topology tree from ASTRAL alongside the fully partitioned and concatenated dataset of all mitochondrial and nuclear genes, with the root of the phylogeny fixed to one. Node labels show the estimated age.

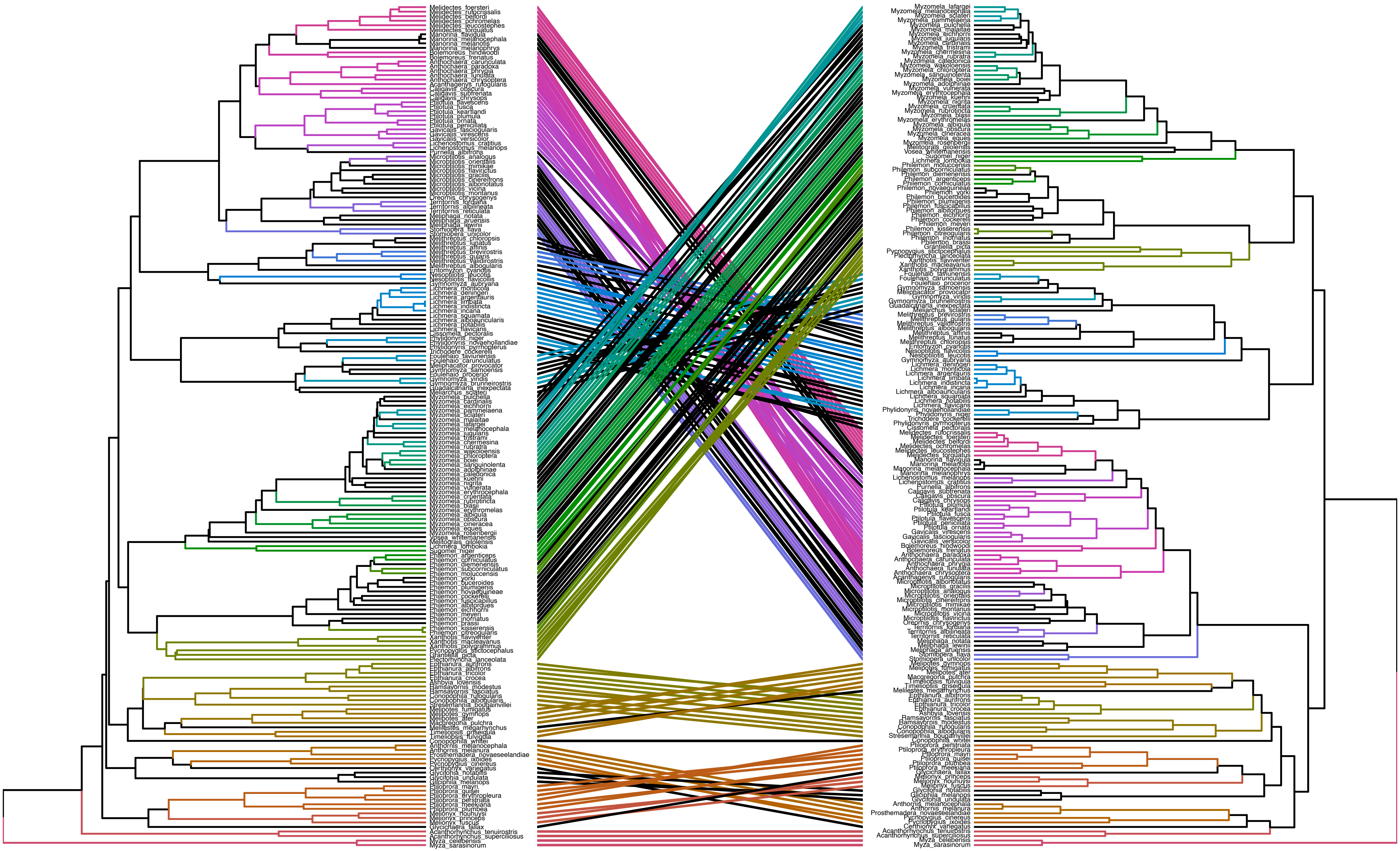

Figure S4. Tanglegram comparing the topology of the phylogeny generated in this study (left) with that obtained from previous studies (right; Marki et al. 2017). Colours indicate similarities and the black lines indicate topological differences, with the lines matching the same species between the two trees. Note the Marki et al. (2017) phylogeny contains 186 honeyeater species and whereas the tree from this study contains 192 species, but here it has been trimmed to the same 186 species.

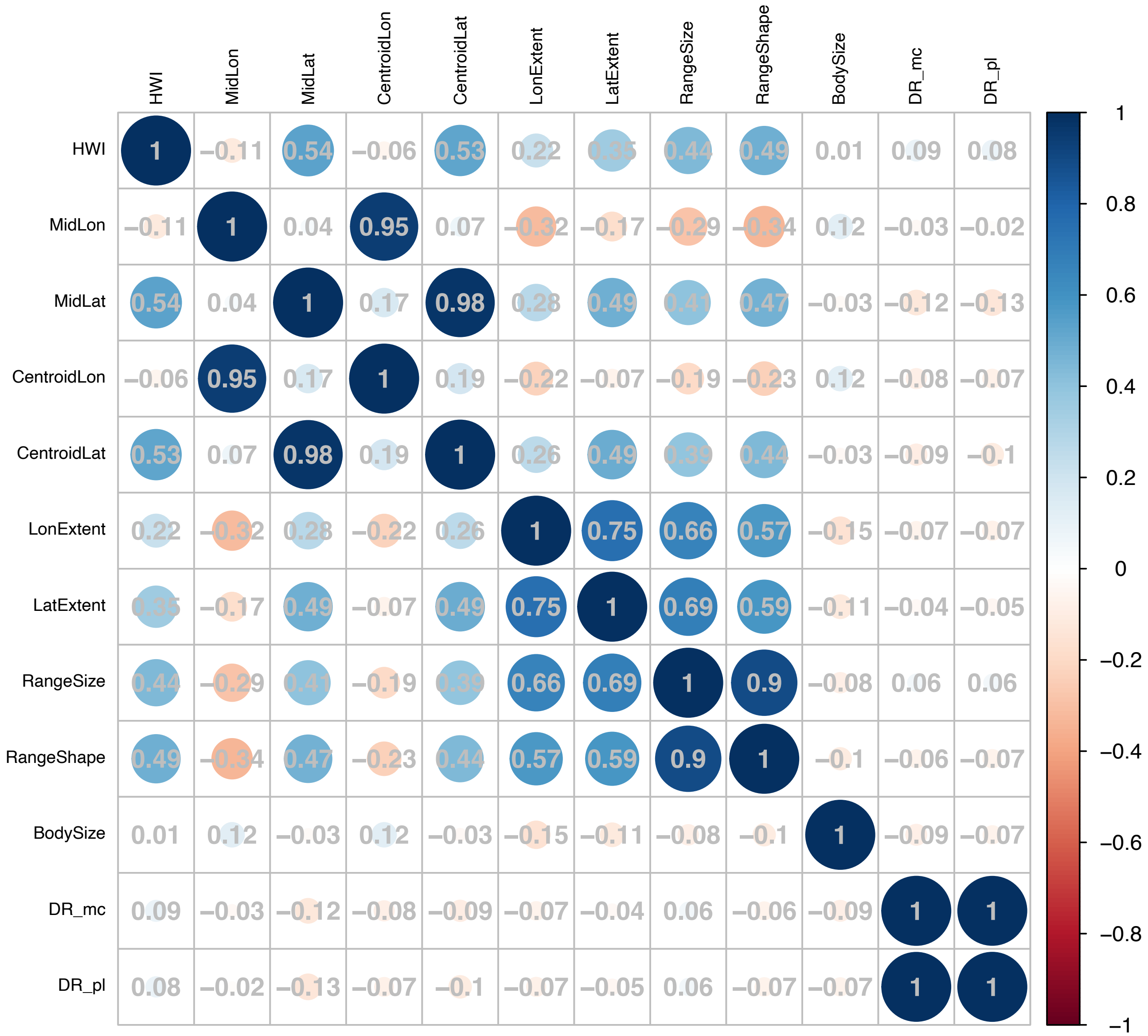

Figure S5. Correlation plot indicating relationships between all traits and range size variables included in this study.

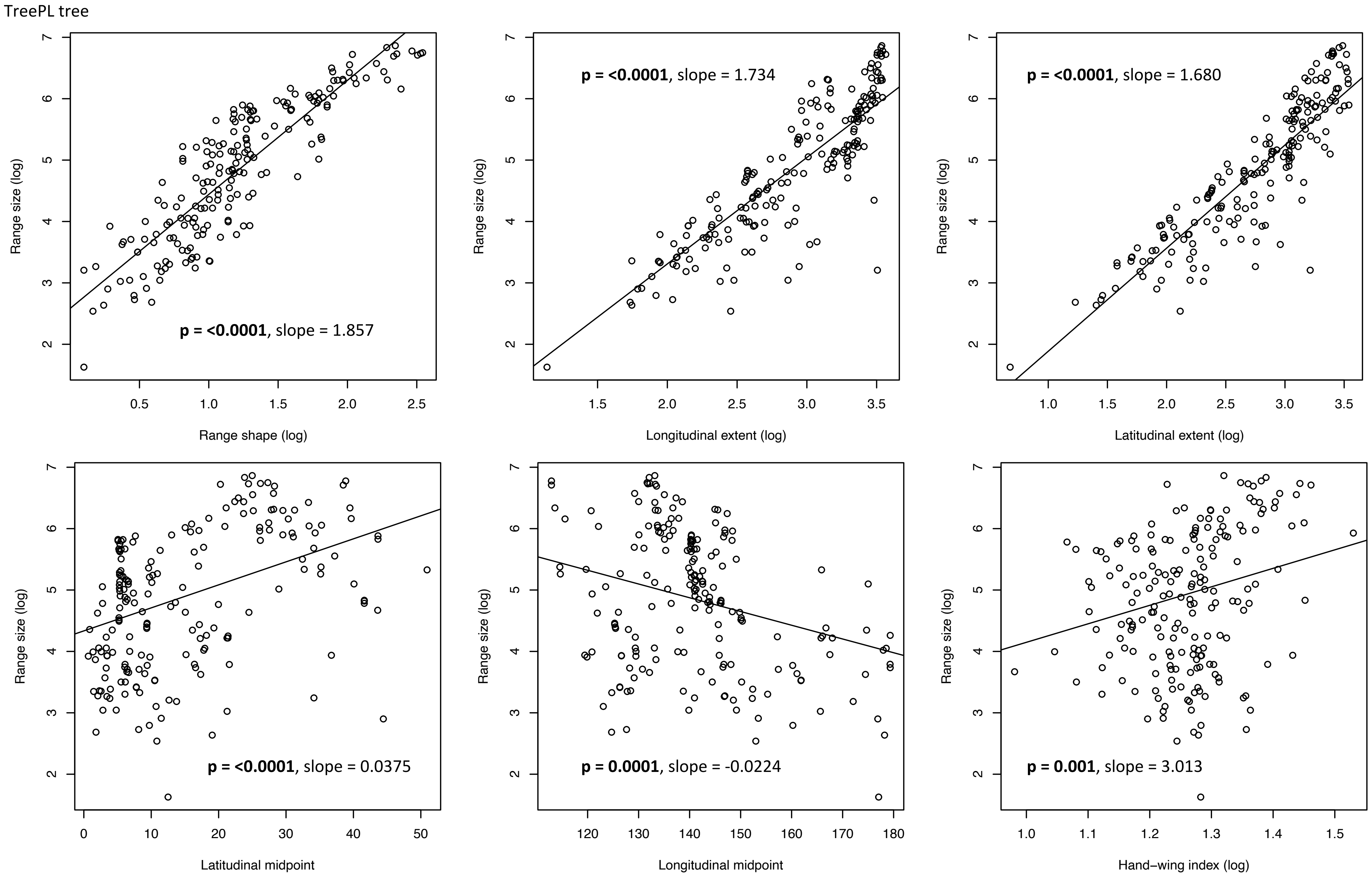

Figure S6. Biplots showing the relationship between traits and variables included in this study. Trendlines and p values are generated from PGLS regressions, using the tree from the TreePL calibration method.

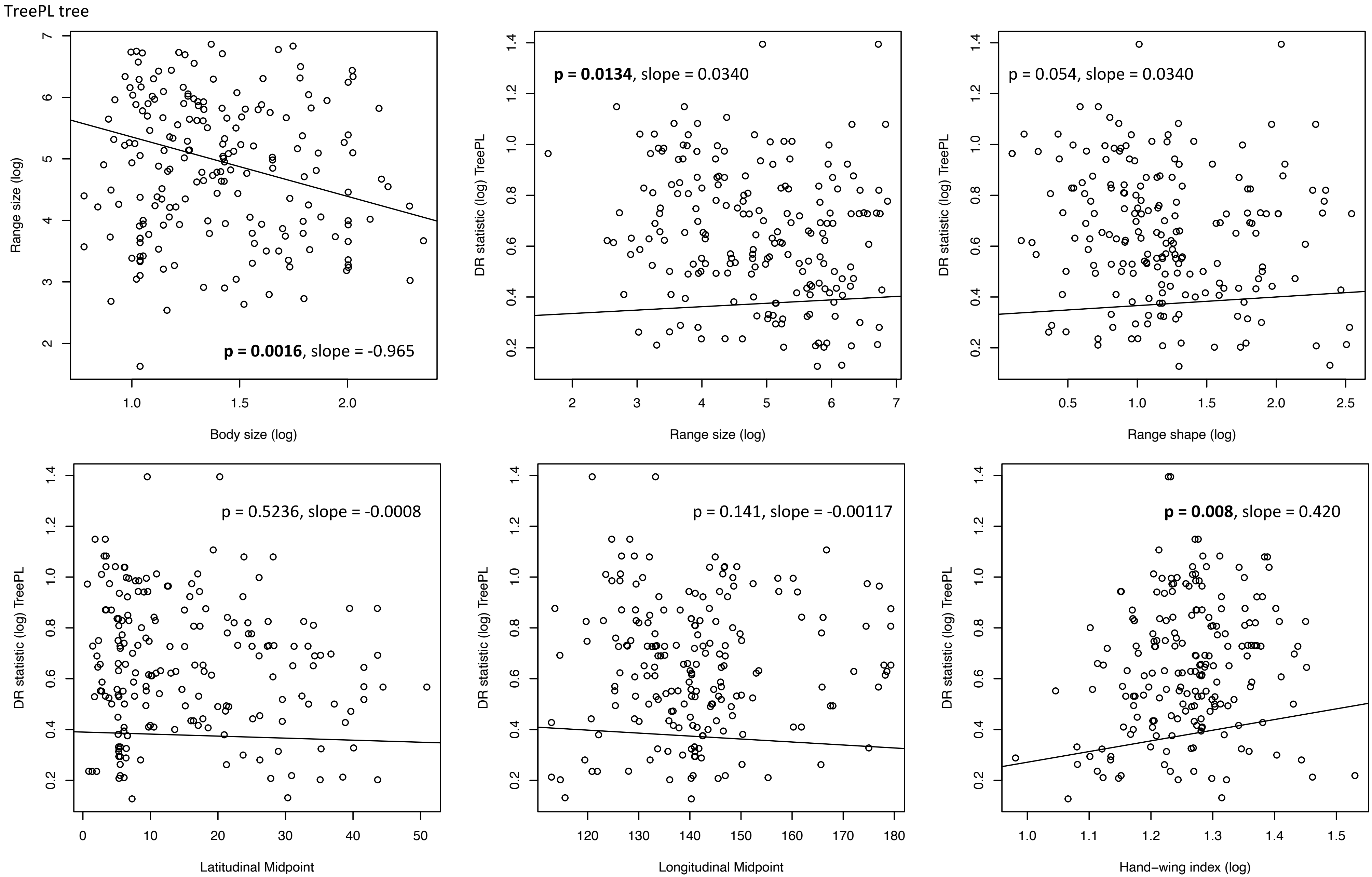

Figure S7. Biplots showing the relationship between traits and variables included in this study. Trendlines and p values are generated from PGLS regressions, using the tree from the TreePL calibration method.

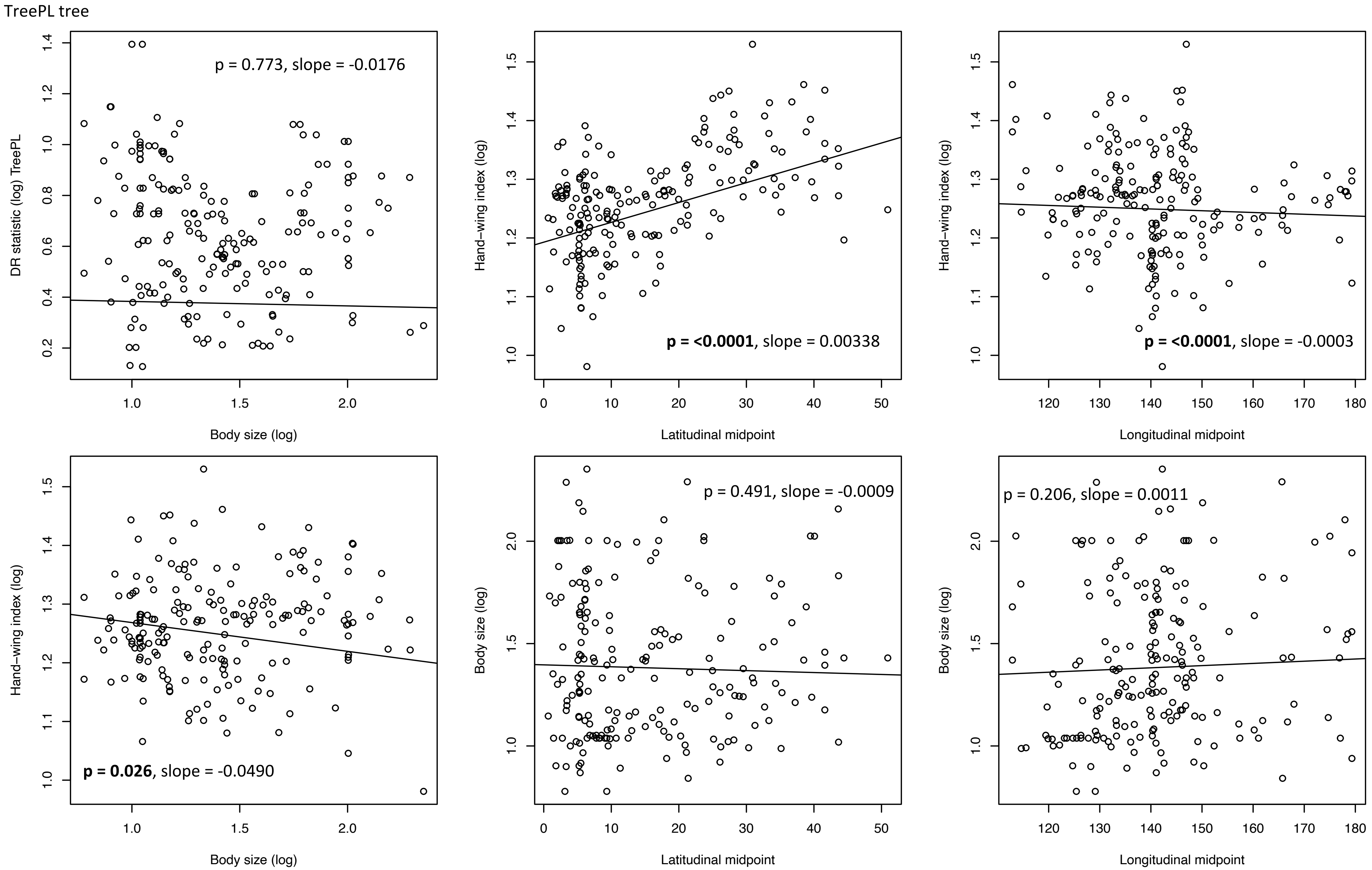

Figure S8. Biplots showing the relationship between traits and variables included in this study. Trendlines and p values are generated from PGLS regressions, using the tree from the TreePL calibration method.

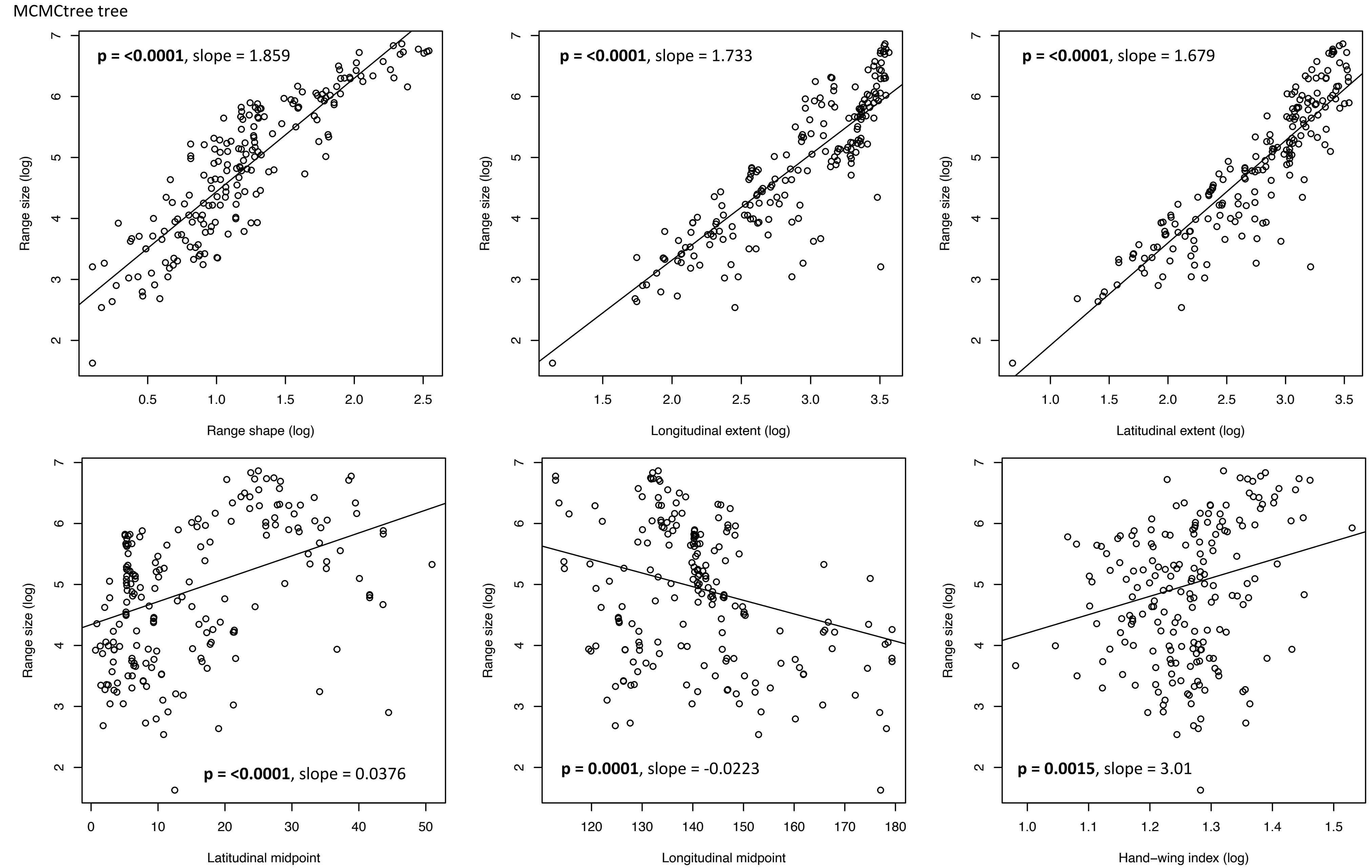

Figure S9. Biplots showing the relationship between traits and variables included in this study. Trendlines and p values are generated from PGLS regressions, using the tree from the MCMCtree calibration method.

MCMCtree tree

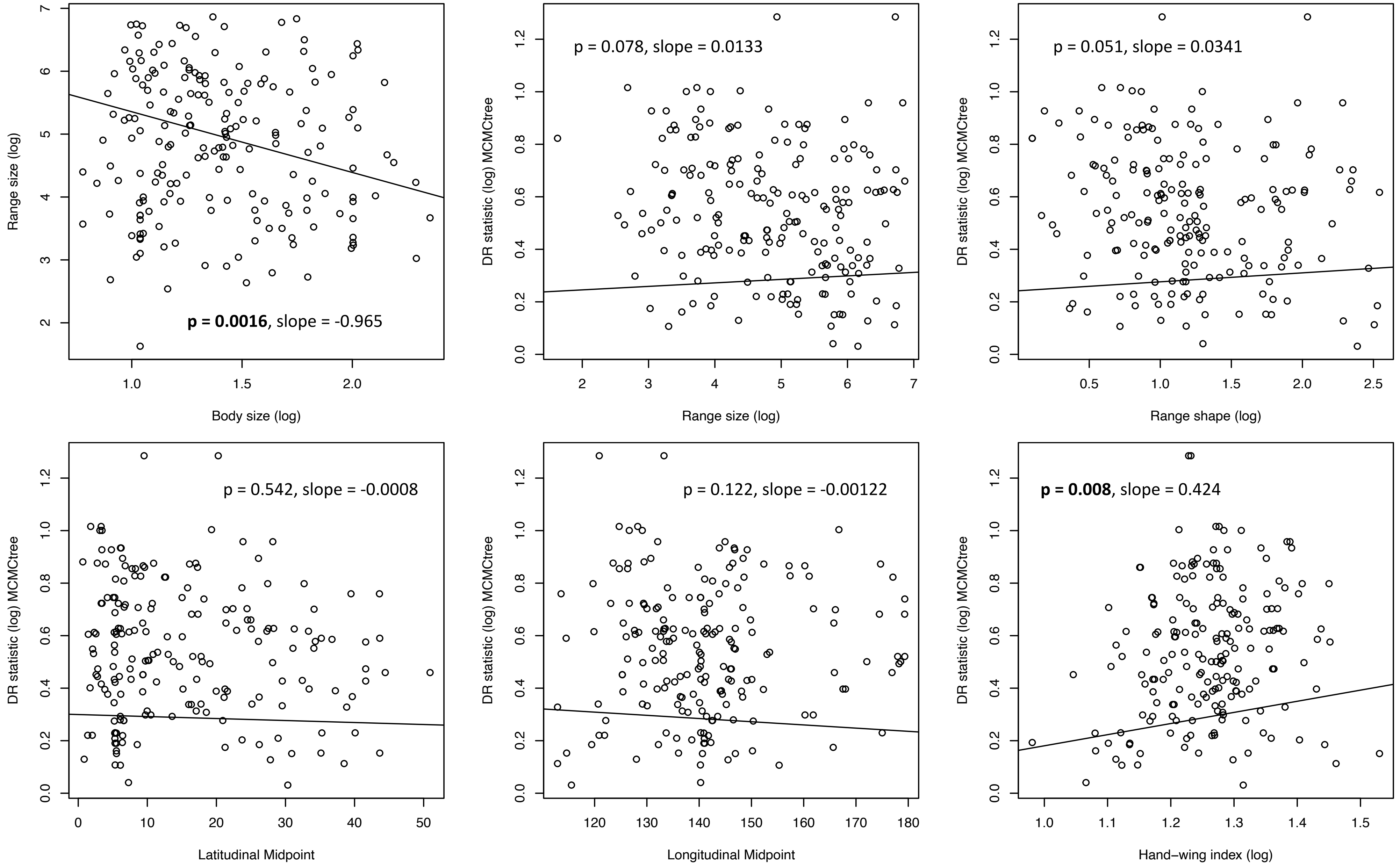

Figure S10. Biplots showing the relationship between traits and variables included in this study. Trendlines and p values are generated from PGLS regressions, using the tree from the MCMCtree calibration method.

MCMCtree tree

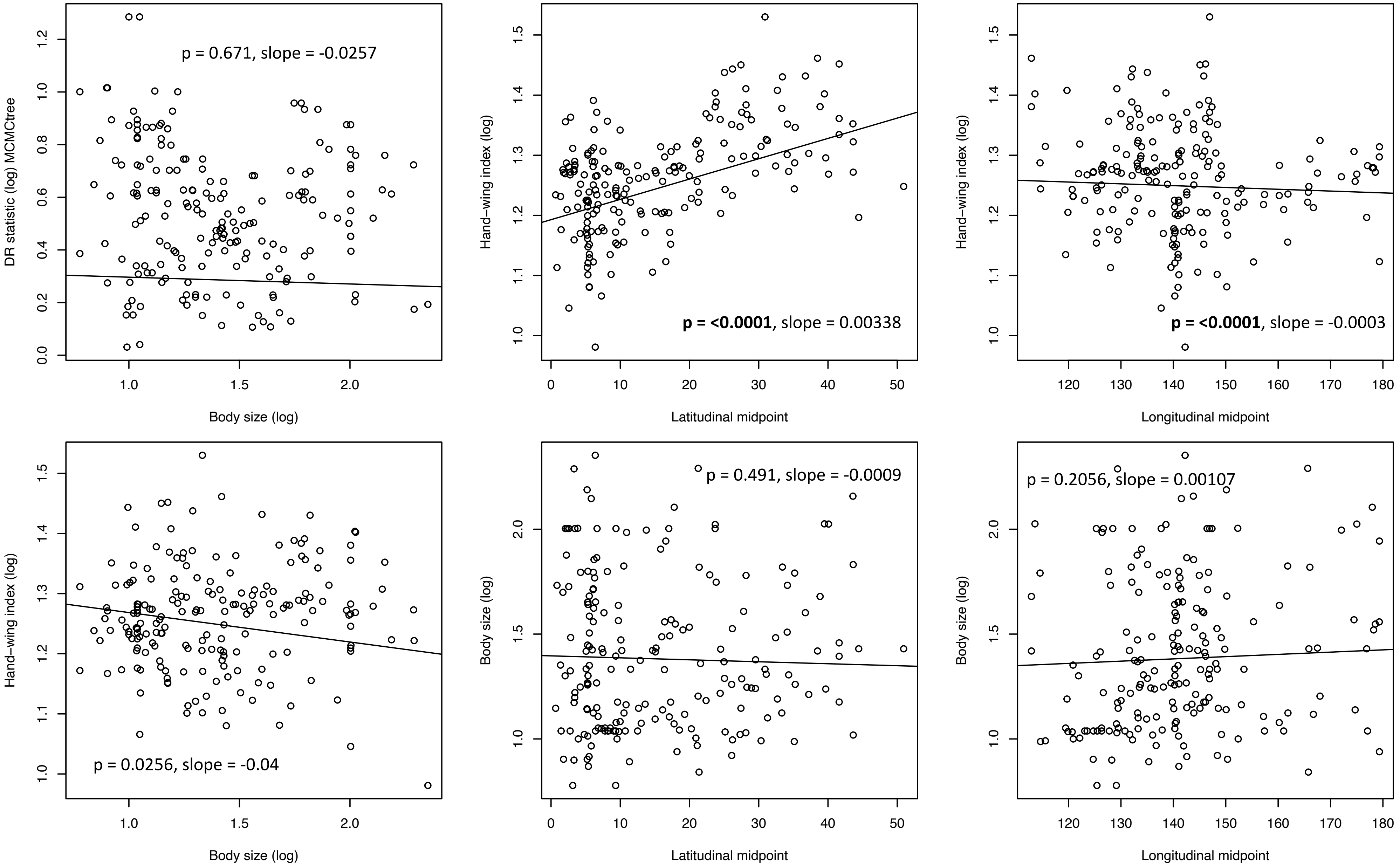

Figure S11. Biplots showing the relationship between traits and variables included in this study. Trendlines and p values are generated from PGLS regressions, using the tree from the MCMCtree calibration method.

TreePL tree

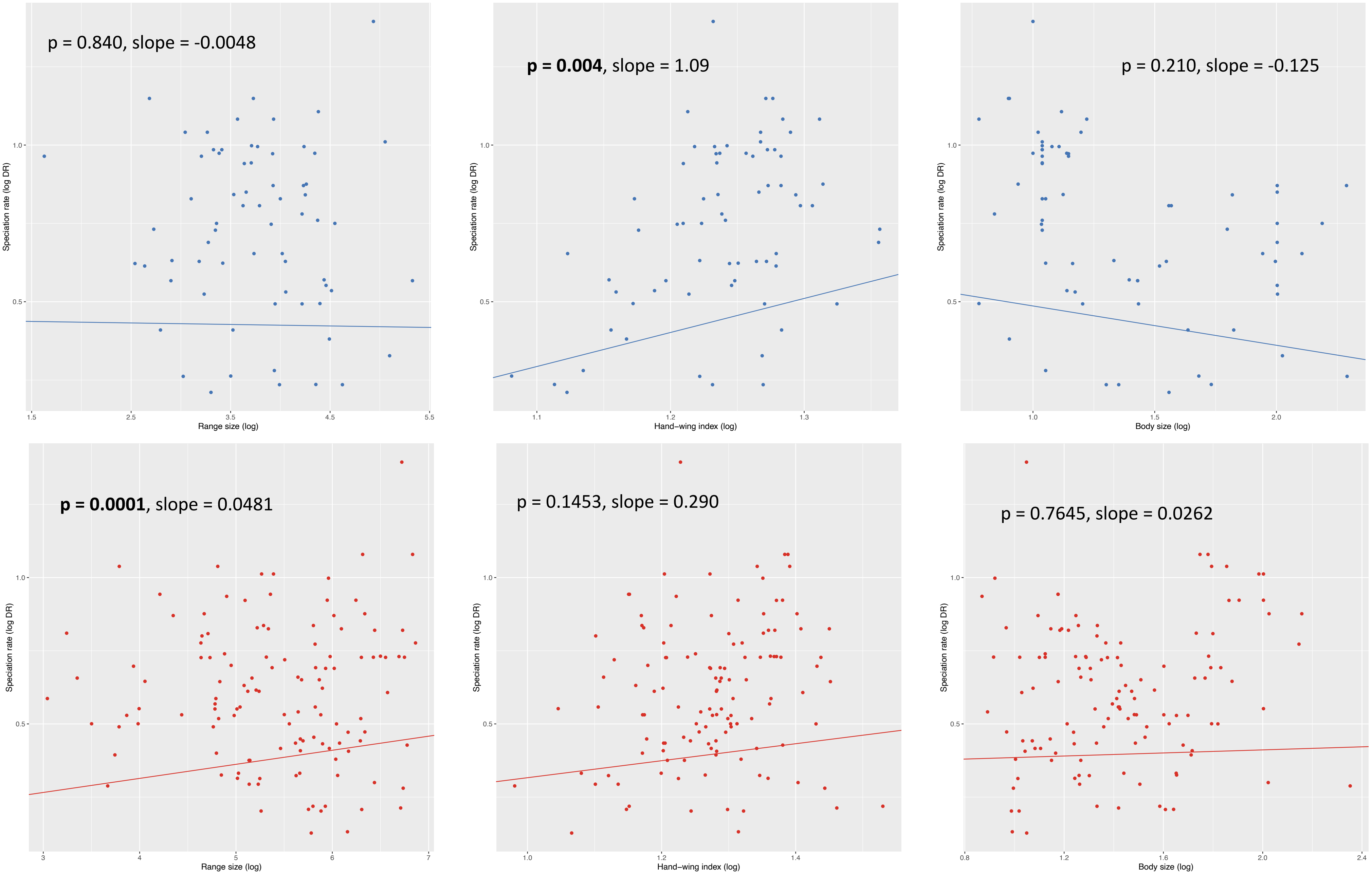

Figure S12. Results from PGLS regressions showing the relationship between traits and variables included in this study, subset into island species (n = 68; top row – blue panels) and continental species (n = 124; bottom row – red panels). Trendlines and p values are generated from PGLS regressions, using the tree from the TreePL calibration method.

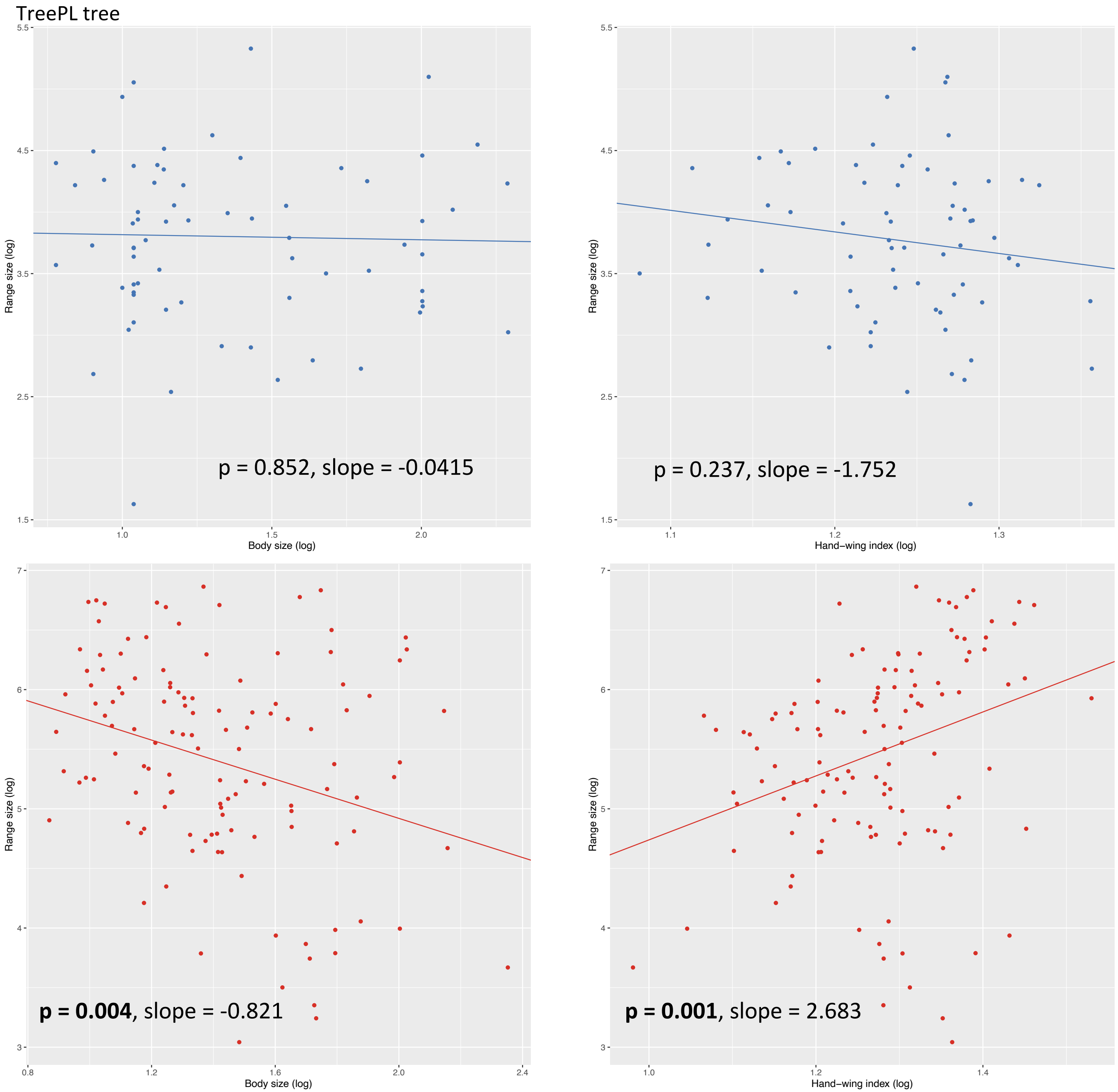

Figure S13. Results from PGLS regressions showing the relationship between traits and variables included in this study, subset into island species (n = 68; top row – blue panels) and continental species (n = 124; bottom row – red panels). Trendlines and p values are generated from PGLS regressions, using the tree from the TreePL calibration method.

MCMCtree tree

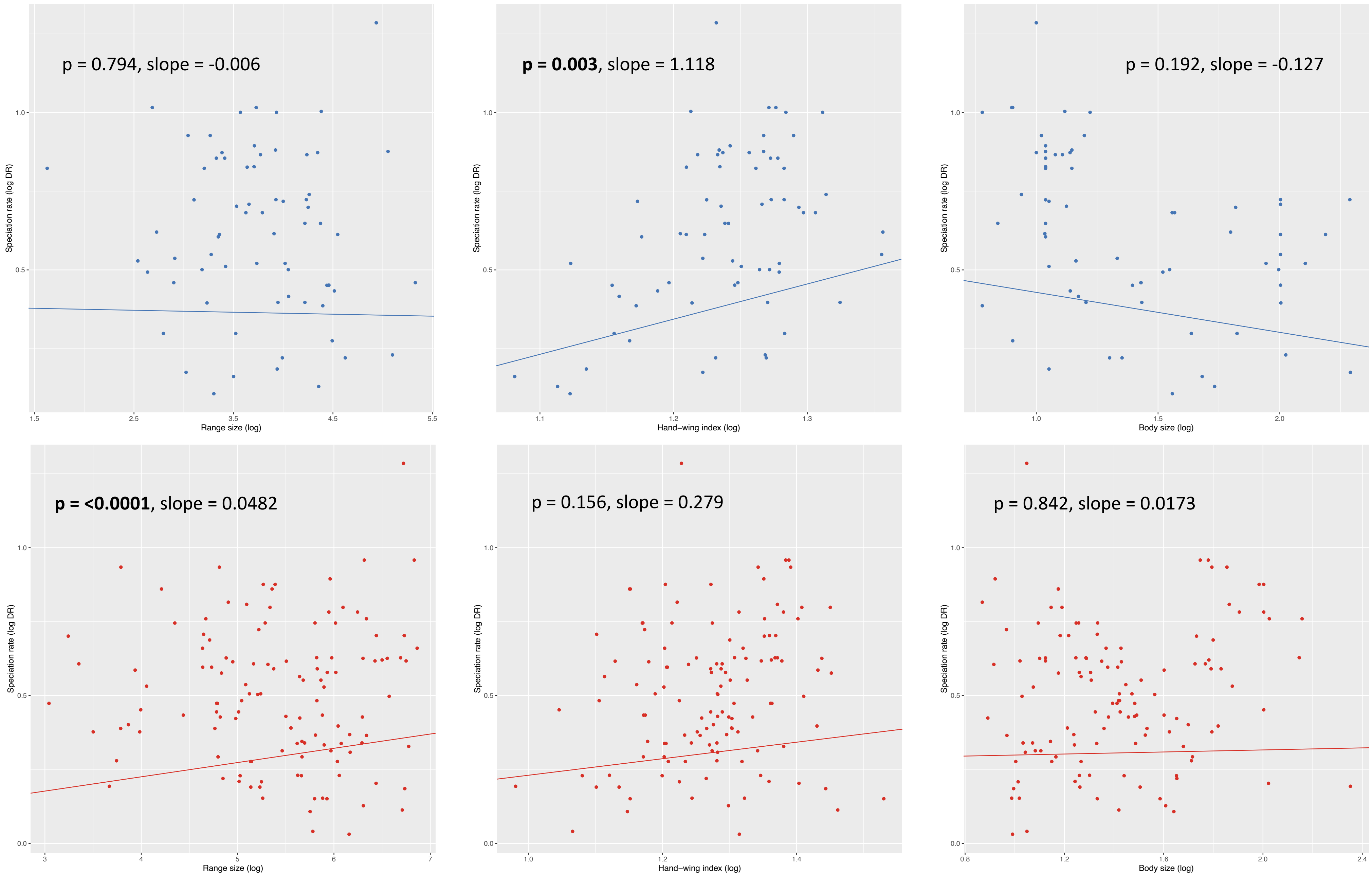

Figure S14. Results from PGLS regressions showing the relationship between traits and variables included in this study, subset into island species (n = 68; top row – blue panels) and continental species (n = 124; bottom row – red panels). Trendlines and p values are generated from PGLS regressions, using the tree from the MCMCtree calibration method.

MCMCtree tree

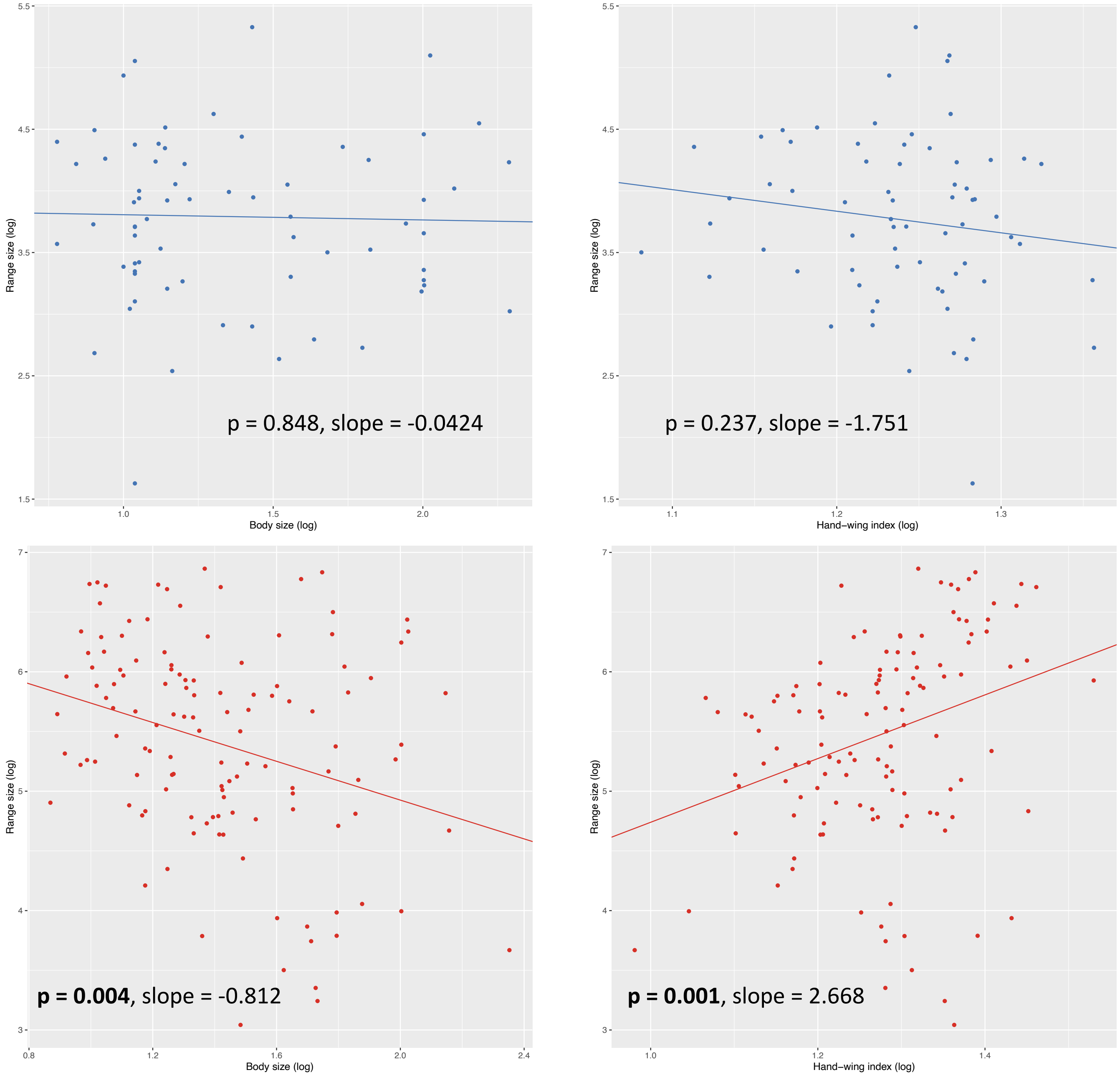

Figure S15. Results from PGLS regressions showing the relationship between traits and variables included in this study, subset into island species (n = 68; top row – blue panels) and continental species (n = 124; bottom row – red panels). Trendlines and p values are generated from PGLS regressions, using the tree from the MCMCtree calibration method.

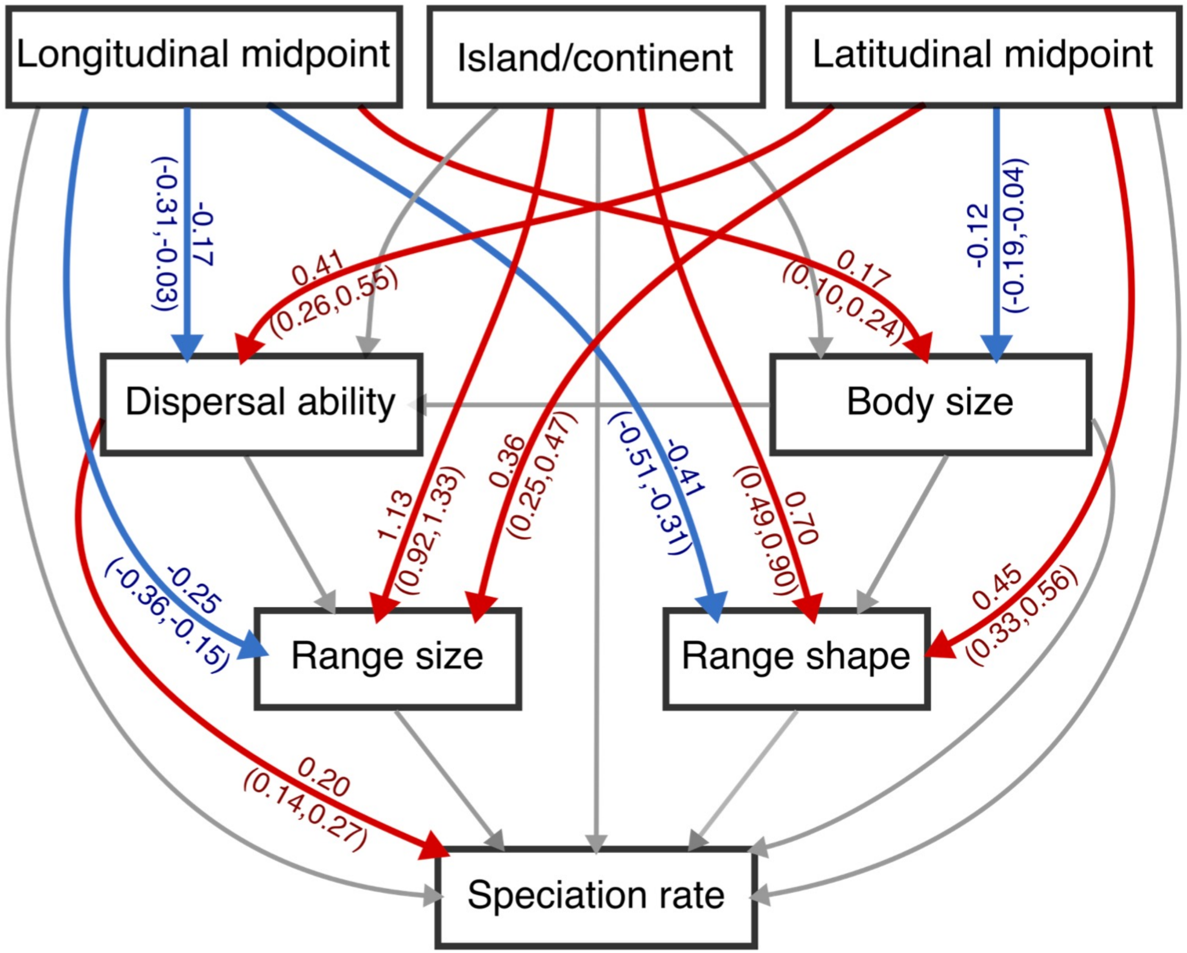

Figure S16. Full phylogenetic Bayesian structural equation model testing the influence of range position (latitudinal and longitudinal midpoints) and islands, on body size and dispersal ability, as well as the interaction between body size and dispersal and the influence of all these traits on range size, range shape, and ultimately speciation. Model results: Blue arrows indicate a significant negative relationship; Red arrows indicate a significant positive relationship. Grey arrows indicate no significant relationship.
