# Supplementary material for: Geographic range size and speciation in honeyeaters
Source: BMC Ecol Evol. 2022 Jun 29;22:86. doi: 10.1186/s12862-022-02041-6 (PMC9245323; doi:10.1186/s12862-022-02041-6)
Supplement: Supplementary file 3 — Additional file 3. Supplementary text file. [file 12862_2022_2041_MOESM3_ESM.docx]

**Additional file 3**

**Geographic range size and speciation in honeyeaters**

by

Eleanor M. Hay, Matthew D. McGee, Steven L. Chown

**Additional Results**

*Phylogenetic reconstruction*

The two calibration methods resulted in very similar phylogenetic trees. Both trees have identical topology, but the TreePL tree has a greater distance between the two *Myza* species and the rest of the honeyeaters (Fig. 2; Fig. S2), whereas the MCMCtree calibration method did not show early divergence of this clade and on average has older node ages for the majority of species (Fig. S3). All analyses were conducted on both trees and results were very similar for both trees (Fig. S6-S15; Tables S1-S10). The treePL tree is presented throughout because the relative placement of the nodes in this tree is more consistent with previous studies that have showed early divergence of *Myza* in comparison to the rest of the honeyeaters [1]. The results from the MCMCtree method are available in Additional tables.

*Speciation rate estimates and range variables*

Range size and all measures of range shape (shape, longitudinal extent and latitudinal extent) and position (estimates for range position using centroids and midpoints) were highly correlated (Fig. S5). Dispersal ability was also highly correlated with all aspects of range size and shape (Fig. S5).

**Additional Methods**

*Phylogenetic inference*

The resulting ASTRAL topology tree (Fig. S1) was used as input for two different calibration methods. For this we used a penalized likelihood implemented in the program treePL [2,3], and a Bayesian method implemented in MCMCtree [4]. Unfortunately, fossil coverage of the honeyeaters is poor; only one honeyeater fossil is known [5]. Since the primary hypotheses being examined in this study do not require accurate dating of the tree, this was not done. Rather the root of the phylogeny was fixed to one for both calibration methods.

For the penalised likelihood approach, we first needed to estimate branch lengths of the tree in substitutions per site. To do so we used RAxML-NG [6]. Here, we used the ASTRAL topology tree alongside the fully concatenated alignment of all 13 nuclear and mitochondrial loci to optimize branch lengths and free model parameters on a fixed topology. The concatenated alignment was partitioned by loci and codon position, and we assigned a separate GTR+I model of rate heterogeneity to each locus. The resulting tree with branch lengths in substitutions per site was then calibrated using penalized likelihood implemented in the program treePL [2,3]. TreePL explicitly allows for rate variation across branches but penalises rate differences after cross-validating initial analyses.

We used MCMCtree from the PAML package v.4.8 [4] to generate a timetree and estimate divergence times using a Markov chain Monte Carlo (MCMC) approach. The ASTRAL topology tree was used as input and alongside all 13 nuclear and mitochondrial genes, partitioned by loci and codon position to estimate branch lengths. The root was constrained to 1 and an independent-rates model was used to relax the clock, with default parameter settings and a HKY85 model of evolution. The analysis was run for 20 000 generations, sampling every 10 iterations, and applying a 1% burn-in. Two separate MCMCtree runs were conducted, convergence of chains was checked in Tracer v.1.7.1 [7], and all parameters exceeded 200.

To have a fully sampled phylogenetic tree for all honeyeater species the two missing species, *Melipotes carolae* and *Myzomela dammermani* were added into each phylogeny using TACT [8]. The placement of these species was based on taxonomic suggestions from Birds of the World available through the Cornell Lab of Ornithology [9]. The time-calibrated phylogenies from the two calibration methods (Fig. S2-S3) are identical in topology, each containing 192 honeyeater species, and were used for all analysis.

*Phylogenetic Bayesian structural equation model details*

To set up the analysis all continuous variables were transformed to have a mean of 0 and a standard deviation of 0.5, ensuring independent variables contribute equally to the analysis. Default priors were initially used and then adjusted based on posterior distributions to aid model performance and convergence. STAN implements a Markov chain Monte Carlo (MCMC) sampler which uses techniques based on Hamiltonian Monte Carlo [10]. Models were run for 8 000 MCMC iterations with 4 chains and convergence was assessed by ensuring the estimated potential scale reduction statistic Rhat was below 1.05 [11]. Effects were judged as significant if the 95% credible intervals of the effect size did not overlap with zero.

*Honeyeater occurrence records*

To model the spatial distributions of each species for the R-INLA analysis, we downloaded available occurrence records for Meliphagidae from eBird via GBIF [12,13]. Duplicate occurrences were removed in R [14] using the function *unique* on longitude and latitude. Erroneous records were removed if they did not fall within known distributions for species, following the BirdLife international expert range maps [15], and range maps and distribution descriptions from Birds of the World available through the Cornell Lab of Ornithology [16]. Occurrence data were not available for five species of honeyeater; *Anthornis melanocephala, Melipotes carolae, Philemon brassi, Myzomela prawiradilagae,* and *Myzomela chermesina.* For these species we randomly generated five occurrence records within their associated range map (provided in Table S13). Five occurrence records were chosen as all species with missing occurrence data all restricted to small ranges and five records gave a reasonable coverage of the distribution of each species. a total of 254 886 honeyeater occurrence records were used in the analysis.

**References**

1. Marki PZ, Jonsson KA, Irestedt M, Nguyen JM, Rahbek C, Fjeldså J. Supermatrix phylogeny and biogeography of the Australasian Meliphagides radiation (Aves: Passeriformes). Mol Phylogenet and Evol. 2017;107:516-529.
2. Sanderson MJ. Estimating absolute rates of molecular evolution and divergence times: a penalized likelihood approach. Mol Biol Evol. 2002;19:102-109.
3. Smith SA, O'Meara BC. treePL: divergence time estimation using penalized likelihood for large phylogenies. Bioinformatics 2012:28;2689-2690.
4. Yang Z. PAML 4: phylogenetic analysis by maximum likelihood. Mol Biol Evol. 2007;24:1586-1591.
5. Boles WE. Fossil honeyeaters (Meliphagidae) from the Late Tertiary of Riversleigh, north-western Queensland. Emu. 2005;105:21-26.
6. Kozlov AM, Darriba D, Flouri T, Morel B, Stamatakis A. RAxML-NG: a fast, scalable and user-friendly tool for maximum likelihood phylogenetic inference. Bioinformatics 2019;35:4453-4455.
7. Rambaut A, Drummond AJ, Xie D, Baele G, Suchard MA. Posterior summarization in Bayesian phylogenetics using tracer 1.7. Syst Biol. 2018;67:901-904.
8. Chang J, Rabosky DL, Alfaro ME. Estimating diversification rates on incompletely sampled phylogenies: theoretical concerns and practical solutions. Syst Biol. 2020;69:602-611.
9. Billerman SM, Keeney BK, Rodewald PG, Schulenberg TS. Birds of the World. Ithaca, NY, USA: Cornell Laboratory of Ornithology; 2020. Available from: <https://birdsoftheworld.org/bow/home>
10. Hoffman MD, Gelman A. The No-U-Turn Sampler: adaptively setting path lengths in Hamiltonian Monte Carlo. J Mach Learn Res. 2014;15:1593-1623.
11. Carpenter B, Gelman A, Hoffman MD, Lee D, Goodrich B, Betancourt M, et al. Stan: A probabilistic programming language. J Stat Softw. 2017;76:1-32.
12. GBIF.org (13 August 2020) GBIF Occurrence Download <https://doi.org/10.15468/dl.ee29jh>
13. eBird Basic Dataset. Version: EBD_relSep-2020. Ithaca, New York: Cornell Lab of Ornithology; Sep 2020.
14. R Core Team. R: A language and environment for statistical computing. R Foundation for Statistical Computing. 2020. Retrieved from https:// www.R-project.org/
15. HBW and BirdLife International. Handbook of the Birds of the World and BirdLife International digital checklist of the birds of the world. Version 4. 2018.
16. Billerman SM, Keeney BK, Rodewald PG, Schulenberg TS. Birds of the World. Ithaca, NY, USA: Cornell Laboratory of Ornithology; 2020. Available from: <https://birdsoftheworld.org/bow/home>
